# Tweaking the NRF2 signaling cascade in human myelogenous leukemia cells by artificial nano-organelles

**DOI:** 10.1073/pnas.2219470121

**Published:** 2024-05-22

**Authors:** Konstantin M. P. Wolf, Viviana Maffeis, Cora-Ann Schoenenberger, Tamara Zünd, Liron Bar-Peled, Cornelia G. Palivan, Viola Vogel

**Affiliations:** ^a^Laboratory of Applied Mechanobiology, Department of Health Sciences and Technology, ETH Zurich, 8006 Zurich, Switzerland; ^b^Swiss National Centre of Competence in Research, Molecular Systems Engineering, 4002 Basel, Switzerland; ^c^Department of Chemistry, University of Basel, 4002 Basel, Switzerland; ^d^Center for Cancer Research, Massachusetts General Hospital/Department of Medicine, Harvard Medical School, Boston, MA 02129, USA

**Keywords:** antioxidant defense, NRF2 signaling, polymersomes, reactive oxygen species, ROS scavenging

## Abstract

NRF2 (nuclear factor erythroid-2-related factor 2) is a key regulator of genes involved in the cell’s protective response to oxidative stress. Upon activation by disturbed redox homeostasis, NRF2 promotes the expression of metabolic enzymes to eliminate reactive oxygen species (ROS). Cell internalization of peroxisome-like artificial organelles that harbor redox-regulating enzymes was previously shown to reduce ROS-induced stress and thus cell death. However, if and to which extent ROS degradation by such nanocompartments interferes with redox signaling pathways is largely unknown. Here, we advance the design of H_2_O_2_-degrading artificial nano-organelles (AnOs) that exposed surface-attached cell penetrating peptides (CPP) for enhanced uptake and were equipped with a fluorescent moiety for rapid visualization within cells. To investigate how such AnOs integrate in cellular redox signaling, we engineered leukemic K562 cells that report on NRF2 activation by increased mCherry expression. Once internalized, ROS-metabolizing AnOs dampen intracellular NRF2 signaling upon oxidative injury by degrading H_2_O_2_. Moreover, intracellular AnOs conferred protection against ROSinduced cell death in conditions when endogenous ROS-protection mechanisms have been compromised by depletion of glutathione or knockdown of NRF2. We demonstrate CPP-facilitated AnO uptake and AnO-mediated protection against ROS insults also in the T lymphocyte population of primary peripheral blood mononuclear cells from healthy donors. Overall, our data suggest that intracellular AnOs alleviated cellular stress by the on-site reduction of ROS.

## Significance

Elevated levels of reactive oxygen species (ROS) are at the core of several chronic inflammatory pathologies, including fibrosis and neurodegenerative disorders. The NRF2 signaling axis is a predominant player in restoring redox homeostasis inside cells. Exploiting NRF2 activation for reboosting cellular ROS detoxification has been suggested as promising therapeutic strategy in age-related inflammatory diseases. Here, we undertake an alternative approach to expand on the ROS defense by introducing artificial nano-organelles (AnOs) with an advanced design into leukemic K562 cells and dissect how they engage in endogenous redox signaling. The demonstrated modulation of signaling pathways by these AnOs paves the way for the development of adjuvant therapies in situations where NRF2 activation is not sufficiently effective.

Reactive oxygen species (ROS) and other radicals are involved in many basic biological processes, e.g., cell growth and proliferation, and are well recognized for their dual role as beneficial and deleterious species in redox biology (1–3). Accordingly, excessive ROS levels may cause damage to cells by irreversible oxidative modifications of proteins, lipids, and glycans, resulting in aberrant functions and eventually cell death (4). Many pathologies characterized by a chronic inflammatory state are associated with the hyperproduction of ROS, including hydrogen peroxide (H_2_O_2_), such as metabolic disorders (5, 6), cancer, fibrosis, and arthritis (7). On the other hand, low local levels of ROS are essential as second messengers in key redox signaling pathways (8). One key player in the maintenance of redox homeostasis is the NRF2 (nuclear factor erythroid-2-related factor 2) signaling pathway. NRF2 is a transcription factor whose nuclear translocation is induced by elevated ROS (9). Under homeostatic conditions, cytoplasmic NRF2 is bound to its negative regulator KEAP1 (Kelch-like ECH-associated protein 1), which targets NRF2 for proteasomal degradation through Cullin-3-induced ubiquitination (10, 11). Cysteines in KEAP1 act as stress sensors that are modified when exposed to increased ROS or electrophiles (12, 13). Oxidation of KEAP1 by H_2_O_2_ triggers conformational changes that result in stabilization and release of NRF2 (11, 14, 15). Stabilized NRF2 translocates to the nucleus (16), where it regulates transcription by binding to promotor elements known as antioxidant response elements [AREs; (16–18)]. AREs control the expression of a battery of defensive genes, such as detoxifying enzymes, antioxidant proteins, proteases, drug transporters, and enzymes of the ubiquitination machinery that collectively promote cell survival (19). NRF2 can reduce oxidative stress levels by directly controlling ROS formation, e.g., by suppressing the expression of inducible nitric oxide synthase [iNOS; (20)], or by inducing the expression of enzymes that break down H_2_O_2_ (21, 22). Many of these enzymes, such as glutathione peroxidases or glutaredoxins, require glutathione as cosubstrate, and also biosynthesis and regeneration of glutathione are catalyzed by NRF2-regulated enzymes.

While exploiting NRF2 activation for boosting cellular ROS detoxification has been suggested as a promising therapeutic approach (23), solutions involving catalytic nanocompartments to efficiently complement the antioxidant response are still underexplored. An advanced strategy is to engineer artificial organelles, given as supplements and taken up by cells, with the potential to alter intracellular metabolic pathways that would drive disease pathogenesis (24–26). While artificial nano-organelles (AnOs) that coencapsulated detoxifying enzymes inside polymersomes were previously shown to mimic native peroxisomes in the conversion of ROS (27), the goal here was to unravel the involvement of AnOs in tuning a target cell’s own antioxidant defense mechanisms. To be able to follow the fate of AnOs on their journey into cells, we developed AnOs having membranes equipped with an imaging moiety by insertion of either DyLight 633 (DL633) or Atto647N DOPE (A647). Besides the combination of an imaging modality and catalytic activity, the advanced AnO design entailed improving cellular uptake rates by presenting cell-penetrating peptides (CPP) on the AnOs’ surfaces. To accomplish efficient H_2_O_2_ decomposition inside cells, we generated polymersomes from a mixture of amphiphilic diblock copolymers based on poly(di-methyl-siloxane-block-poly(2-methyl-2-oxazoline) instead of only one triblock copolymer as reported for artificial peroxisomes (27). The resulting polymersome membrane is more flexible than PMOXA-*b*-PDMS-*b*-PMOXA derived membranes which is conducive to a rapid insertion of pore-forming melittin (mel) as gate for molecular diffusion (28). In addition, the improved mechanical stability of the membrane based on diblock over triblock copolymers (29–31) is crucial to preserve the polymersome integrity upon simultaneous insertion of permeabilizing and fluorescent entities. A complex scenario of conditions is required to obtain AnOs with both improved uptake capability and integrated imaging modality. In particular, membrane modification with a fluorescent dye and mel requires fine-tuning of the conditions in order to prevent the abrogation of in situ mel pore formation or membrane destabilization. Besides, the dye entrapment should not interfere with the accessibility of the functional azide groups necessary for CPP attachment on the polymersomes. Furthermore, our AnOs contained the H_2_O_2_-degrading biocatalyst lactoperoxidase (LPO) in their cavity, which is free inside the AnOs to perform its activity, yet protected against proteolytic attack from cellular proteases. Their influence on ROS metabolism was demonstrated by using K562 myelogenous leukemia cells engineered to report NRF2 activation and compared with peripheral blood mononuclear cells (PBMCs) with a special focus on T lymphocytes. Under conditions where the endogenous NRF2-activated defense is exhausted, AnOs successfully complemented the cell's existing antioxidant enzyme repertoire and effectively conferred protection against cell death.

## Results

### Design of Fluo-AnOs Metabolizing H_2_O_2_ and Functional Validation.

**Fig. 1.**
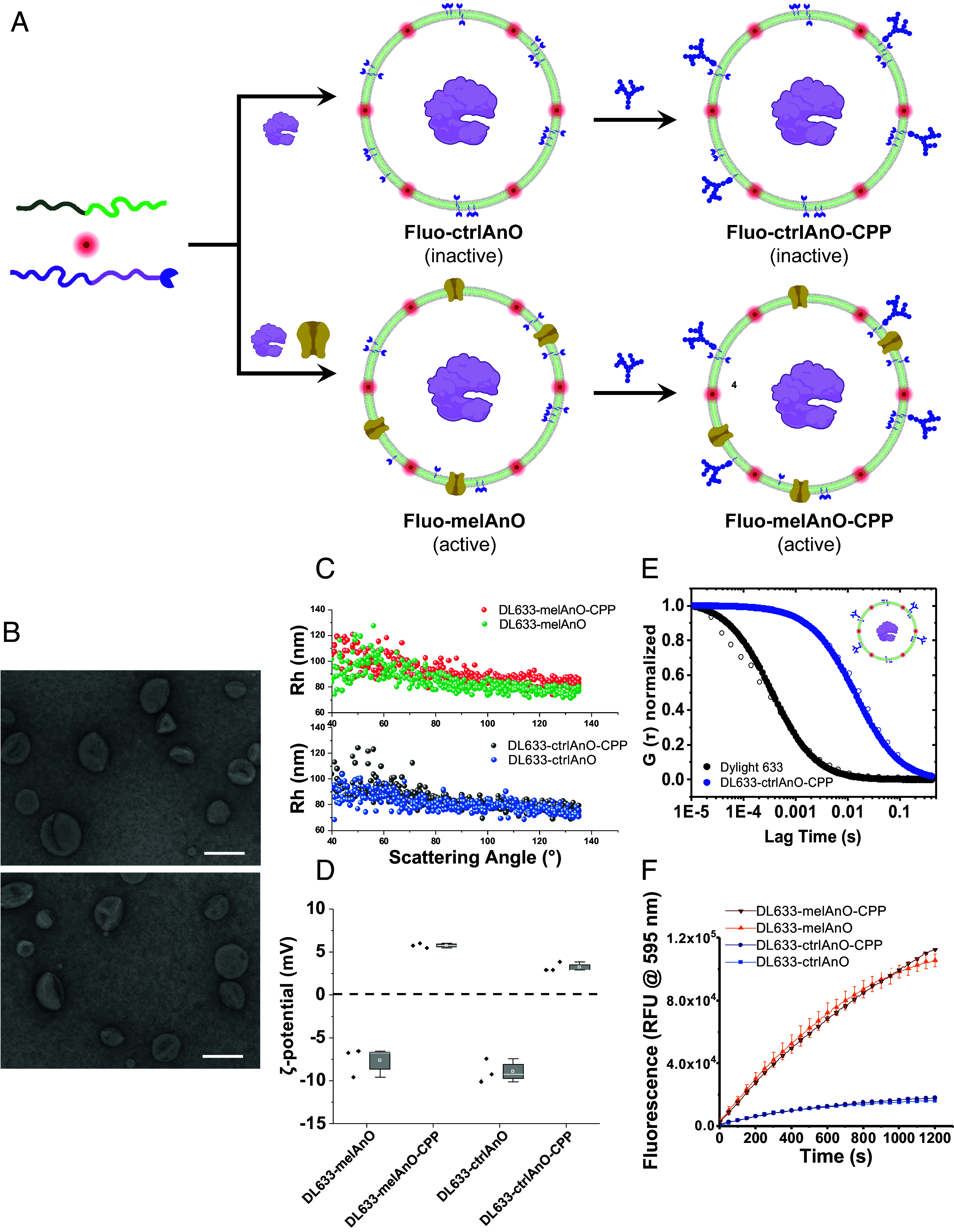
Design, characterization, and functionality of fluo-AnOs. (*A*) Design of different AnOs harboring fluorescent dyes in their membrane and lactoperoxidase (LPO) in their aqueous interior. A mixture of PMOXA_29_-*b*-PDMS_10_ and PDMS_29_-*b*-PMOXA_10_-PEG_4_-N_3_ diblock copolymers was dried together with a fluorescent dye to a thin film which was subsequently induced to self-assemble into polymersomes by rehydration with PBS in the presence of LPO (fluo-AnOs). Melittin was added to the rehydration solution to obtain AnOs with membrane pores (active). Postassembly, AnOs were surface-functionalized with a peptide dendrimer (CPP). (*B*) TEM micrographs of DL633-melAnO-CPP (*Top*) and DL633-ctrlAnO-CPP (*Bottom*) showing the typical morphology of collapsed polymersomes. Scale bar, 100 nm. (*C*) Light scattering measurements over scattering angles from 40° to 130° of active DL633-melAnOs and inactive DL633-ctrlAnOs with and without CPP functionalization. (*D*) ζ-potential measurements of active and inactive DL633-AnOs, nonfunctionalized, and functionalized with CPP. (*E*) Normalized FCS autocorrelation curves (open circles: raw data; filled circles: fitted curves) of free DL633-NHS-ester (black) and DL633 labeled ctrlAnOs-CPP (blue). (*F*) Enzymatic activity of CPP-functionalized DL633-melAnO-CPP (dark red), nonpermeabilized DL633-ctrlAnO-CPP (dark blue), and nonfunctionalized permeabilized DL633-melAnO and nonpermeabilized DL633-ctrlAnO (light red and light blue, respectively) was determined by monitoring the conversion of AmplexRed (AR) to a resorufin-like product (RLP) at 560 nm over a total of 20 min.

To engineer the next generation of ROS degrading AnOs with an integrated tracer modality, here for fluorescence imaging, we first mixed PDMS_29_-*b*-PMOXA_10_ at a 1:1 ratio with PDMS_29_-*b*-PMOXA_10_-PEG_4_-N_3_, whose azide end groups render the surface of assembled AnOs amenable to functionalization [Fig. 1*A*; (24)]. To provide AnOs with an imaging modality, we dried the PDMS_29_-*b*-PMOXA_10_ and PDMS_29_-*b*-PMOXA_10_-PEG_4_-N_3_ copolymer films together with either DyLight 633 (DL633) or Atto647N DOPE (A647) to promote association of the fluorescent dye with the polymersome membrane during self-assembly. Accordingly, thin films were rehydrated in LPO containing PBS without/with melittin as previously reported (32). Introducing a fluorescent label was crucial for rapid visualization of AnOs during uptake and ROS degradation in cells. However, it resulted in a complex set of requirements: i) preserving the integrity of the membranes, ii) not blocking the melittin pores, and iii) not hindering the accessibility of the azide groups, crucial for efficient attachment of CPP. First, the self-assembly process has been optimized by considering combinations of melittin and fluorescent dyes that supported pore formation and preserved AnO integrity. The resulting fluorescently labeled AnOs (fluo-AnOs) were extruded, treated with proteinase K to digest nonincorporated melittin and LPO, and finally purified by size exclusion chromatography to remove all nonincorporated contaminants including free dye. In order to enhance AnO uptake into cells, a dendritic CPP was attached to their surface via DBCO-coupling, resulting in fluo-AnOs with either nonpermeabilized membranes (fluo-ctrlAnO-CPP) or melittin-permeabilized membranes (fluo-melAnO-CPP) [Fig. 1*A*, (33)]. The conditions of CPP attachment used for nonfluorescent AnOs (32) have been adjusted for the fluo-AnOs to achieve a similar surface density of the targeting molecules and to avoid blocking the melittin pores. We selected a dendritic peptide with short dipeptide branches which has been reported to convey higher cell penetration, lower toxicity, and higher serum stability (*SI Appendix*, Fig. S1) (34). As the cellular up-take of nanoassemblies, including polymersomes, depends on the particle size (35), all AnOs were extruded 11 times through 100 nm pore diameter membranes to decrease the size and polydispersity prior to physicochemical and morphological characterization.

A combination of transmission electron microscopy (TEM), light scattering, zeta-potential measurements, and fluorescence correlation spectroscopy (FCS) was used to characterize the fluorescent, active and inactive AnOs without CPP (fluo-melAnOs and fluo-ctrlAnOs, respectively) and with CPP (fluo-melAnOs-CPP and fluo-ctrlAnOs-CPP; Fig. 1). The effect of membrane modifications such as the insertion of permeabilizing or fluorescent moieties and the attachment of targeting moieties on the overall AnO activity was evaluated by comparison to nonfluorescent AnOs.

TEM of negatively stained AnOs revealed the deformed morphology typical for polymersomes for DL633-melAnOs-CPP (Fig. 1 *B*, *Top*) and DL633-ctrlAnOs-CPP (Fig. 1 *B*, *Bottom*). This indicates that the addition of the fluorescent dye either alone or in combination with melittin did not hinder the formation process of AnOs. The TEM micrographs showed no signs of AnO aggregation, which indicates that the dye molecules were entrapped in the synthetic membrane. This is in agreement with the light scattering results where the hydrodynamic radius, Rh, of all fluorescent AnOs remained stable over scattering angles ranging from 40° to 130° (Fig. 1*C*). In addition, the Rh values for the fluorescent AnOs were similar to those reported for nonfluorescent AnOs (32). The flexibility of the membranes allowed also polymersomes with slightly higher Rh to pass through the pores of the extrusion membranes (36). Zeta potential measurements indicate an increase in surface charge from −7.6 ± 1.6 mV for DL633-melAnOs to 5.7 ± 0.2 mV for CPP-decorated DL633-melAnOs (Fig. 1*D*). Similarly, the surface charge of the nonpermeabilized AnOs increased from −8.9 ± 1.3 mV to 3.2 ± 0.6 mV in CPP-decorated DL633-ctrlAnOs. The change of the zeta-potential values reflects the attachment of CPP.

The labeling of the membrane with DL633 was further characterized by FCS (Fig. 1*E* and *SI Appendix*, Tables S1 and S2). In addition, as the DL633 fluorophore primarily reacts with the terminal OH-groups of the hydrophilic PMOXA domain, we alternatively tested Atto 647 N DOPE (A647; *SI Appendix*, Fig. S2), a hydrophobic dye prone to integrate into the hydrophobic PDMS block. Any change in the architecture of AnOs, including the newly introduced fluorescent tracking moiety, might influence the AnOs’ enzymatic activity and/or uptake dynamics in cells, which is why we selected two dyes with sufficiently different chemistries to be able to generalize our findings. FCS probes the Brownian motion of a fluorescent species in a femto-liter sized volume, yielding molecular parameters including the diffusion time and the number of particles, thus serving to assess interactions/entrapment of the fluorescent species with/in supramolecular assemblies. By fitting the autocorrelation curves, we obtained as average diffusion times, τ_D_ = 53 μs for free DL633 and τ_D_ = 4,231 ± 1 ms for DL633-ctrlAnO-CPP (Fig. 1*E* and *SI Appendix*, Table S1). The significantly higher τ_D_ for DL633-ctrlAnO-CPP indicated successful labeling of the membrane. Further, based on the brightness measurements, we quantified how many dye molecules on average were present per AnO (*SI Appendix*, Table S2). The value of the molecular brightness of DL633-ctrlAnOs-CPP (expressed as counts per molecule, CPM) was divided by the CPM of freely diffusing DL633-NHS-ester, yielding an average of 88 ± 10 dye molecules per AnO (*SI Appendix*, Table S1). In addition, the brightness for DL633-ctrlAnO and DL633-melAnO (CPM 388 and 382, respectively) was similar to the values obtained for DL633-ctrlAnO-CPP and DL633-melAnO-CPP (CPM 406 and 399, respectively), suggesting that melittin and CPP functionalization does not lead to DL633 bleaching (*SI Appendix*, Table S2). Correspondingly, FCS revealed membrane insertion of 274 ± 8 dye molecules per AnO in A647-ctrlAnOs-CPP (*SI Appendix*, Fig. S2 and Table S1), with no bleaching effect when melittin was coinserted in the synthetic membrane (*SI Appendix*, Table S2).

We next assessed the LPO content inside fluorescent and nonfluorescent AnOs by a bicinchoninic assay, a copper-based colorimetric assay for total protein quantification. The LPO concentration of 30 μg mL^−1^ obtained for nonfluorescent AnOs corresponds to an encapsulation efficiency of 33 ± 10%, which agrees with the values of 27 μg mL^−1^ obtained for the corresponding fluorescent AnOs. These encapsulation values are in the normal range reported for encapsulation of other enzymes in PDMS-*b*-PMOXA polymersomes (25, 37, 38).

The bulk catalytic activity of DL633-AnOs, both nonpermeabilized (DL633-ctrlAnOs) and permeabilized (DL633-melAnOs), was evaluated fluorometrically by measuring the equimolar in situ conversion of AmplexRed (AR) to a fluorescent resorufin-like product (RLP) upon consumption of H_2_O_2_ (Fig. 1*F*). Melittin pores allow both H_2_O_2_ and AR to diffuse into the AnO cavity where AR is oxidized by LPO in the presence of H_2_O_2_. Accordingly, the enzymatic activity of LPO was significantly higher when the AnO membrane was rendered permeable by melittin (DL633-melAnOs) compared to the very low activity of nonpermeabilized AnOs (DL633-ctrlAnOs). The latter most likely represents AR autoxidation as residual nonencapsulated LPO outside AnOs was removed by proteinase K treatment during AnO preparation. Furthermore, comparison of enzyme kinetics of permeabilized DL633-AnOs with and without surface-attached CPPs showed that CPP-functionalized DL633-melAnOs were equally functional. This indicates that neither the labeling of the AnO membrane nor the CPPs exposed at the external interface affected the LPO activity. Small differences in fluorescence production over time are likely due to the higher melAnO-CPP concentration as obtained by Nanoparticle Tracking Analysis (NTA) measurements (*SI Appendix*, Table S3). Similarly, the activity of A647-AnOs was only marginally affected by the dye insertion and/or the CPP attachment (*SI Appendix*, Fig. S3).

### Construction of an NRF2 Activity Reporter Cell Line.

**Fig. 2.**
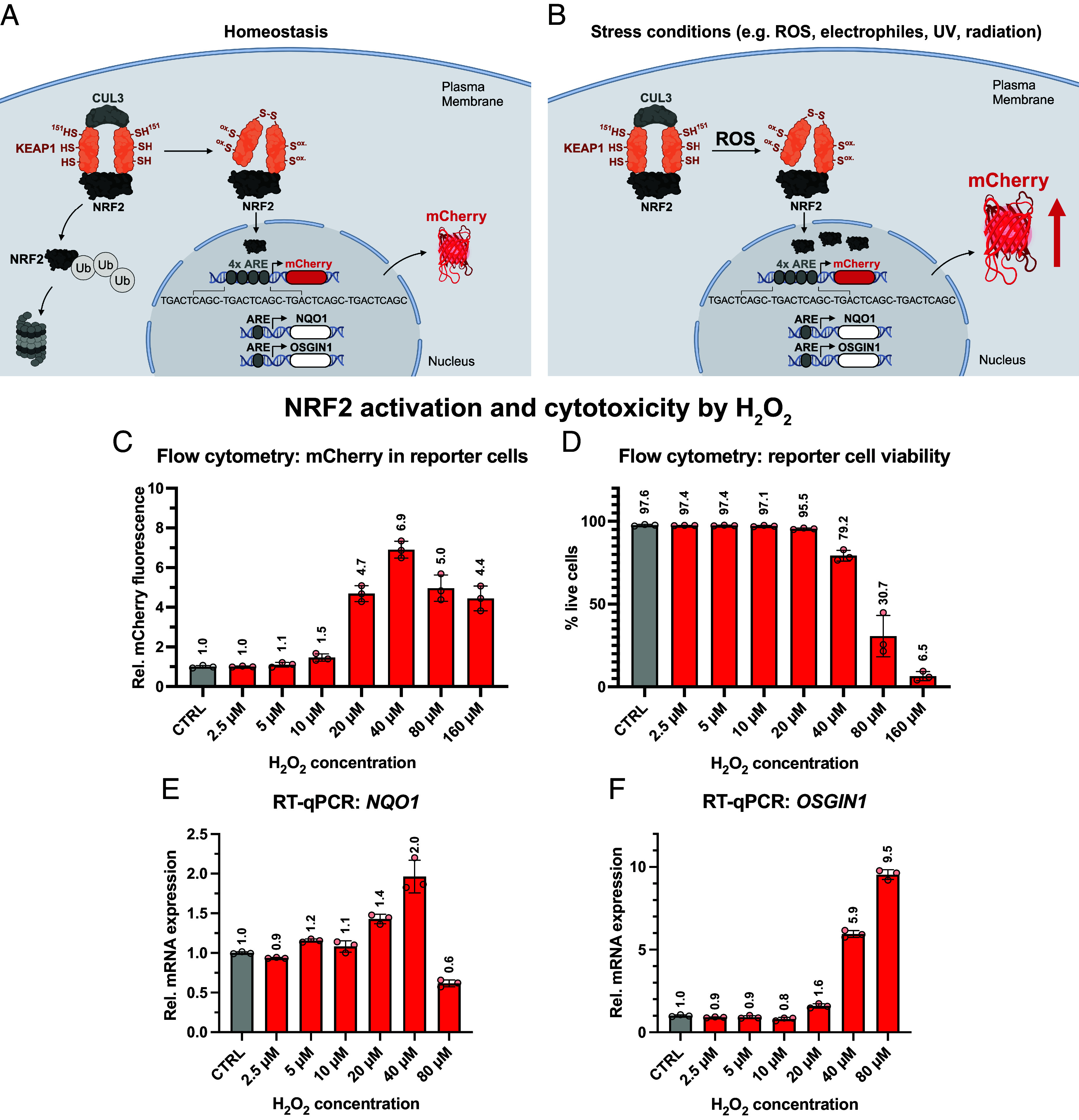
Reporter cells to probe NRF2 transcriptional activation upon H_2_O_2_ exposure. Schematic of the reporter cell. (*A*) Under homeostatic conditions, KEAP1 binds to NRF2 and marks it for proteasomal degradation. Because of low NRF2 abundance in the nucleus, AREs are not occupied and mCherry expression and expression of endogenous NRF2 target genes, *NQO1* and *OSGIN1*, is low. (*B*) In stressed cells, ROS can induce a conformational change of KEAP1 that releases NRF2. Free NRF2 translocates to the nucleus and initiates transcription of mCherry and endogenous NRF2 target genes by binding to AREs. NRF2 activation and cytotoxicity by H_2_O_2_. (*C*) NRF2 activation in reporter cells was examined by flow cytometry upon treatment with different H_2_O_2_ concentrations (without or with 2.5 to 160 µM H_2_O_2_). mCherry fluorescence levels per cell were quantified and normalized to constitutive CMV-promoter-driven EGFP expression (triplicates with *N* = 5,000 events each, error bars = SD). (*D*) Cell viability was determined via violet LIVE/DEAD™ stain using flow cytometry. Expression levels of endogenous NRF2 target genes, *NQO1* in (*E*), *OSGIN1* in (*F*), upon exposure to varying levels of H_2_O_2_ (without or with 2.5 to 80 µM H_2_O_2_) in reporter cells were determined by RT-qPCR and normalized to global 18S rRNA levels. Mean of plotted values is indicated above bars; error bars indicate SDs of technical triplicates (N = 3, error bars = SD).

To quantify intracellular ROS load, we generated a reporter cell line based on K562 myelogenous leukemia cells that can inform on the NRF2 activation state. In these cells, we introduced a previously described genetic cassette by lentiviral infection encoding mCherry downstream of a minimal promotor regulated by four upstream AREs (Fig. 2 *A* and *B*) (39, 40). The ARE sequence used in the construct, TGACTCAGC, corresponds to that of the endogenous NRF2 target gene NAD(P)H quinone dehydrogenase 1 (*NQO1*) (41) and was separated by spacer sequences between each repeat. The entire cassette was cloned into a vector with flanking long terminal repeats for stable lentiviral gene delivery to K562 cells. To report protein expression independent of NRF2 regulation, we coinfected K562 cells with lentivirus encoding EGFP under control of a constitutively active cytomegalovirus (CMV) promoter. In these reporter cells, H_2_O_2_ induced NRF2 activity is revealed by mCherry fluorescence and/or by quantifying transcription of endogenous NRF2 target genes, such as *NQO1* or *OSGIN1* (oxidative stress induced growth inhibitor 1), as summarized for unstressed condition (Fig. 2*A*) and upon stress (Fig. 2*B*).

To test the ROS sensitivity of the reporter cells, we exposed them to increasing levels of H_2_O_2_ (0 to 160 µM) and assessed the NRF2 activation by quantifying mCherry fluorescence using flow cytometry (Fig. 2*C*). Increasing H_2_O_2_ concentrations induced mCherry expression to a maximum of 6.9-fold at 40 µM H_2_O_2_ compared to untreated cells. To assess whether the substantially lower mCherry expression at 80 µM and 160 µM was related to an increasing cytotoxicity at these high H_2_O_2_ concentrations, we determined cell viabilities using ThermoFisher’s violet LIVE/DEAD™ stain (Fig. 2*D*). Cell viability was around 95 to 97% for concentrations up to 20 µM H_2_O_2_, but started to decrease at higher concentrations with less than 10% viable cells at the highest concentration of 160 µM, where NRF2 activation could not be reliably quantified anymore.

To explore the sensitivity of the reporter to H_2_O_2_ compared to endogenous NRF2 target genes, which are regulated by a single ARE, rather than by a repeat of 4× AREs, and to validate that the reporter physiologically reflects NRF2 activity, we further measured *NQO1* and *OSGIN1* expression in response to H_2_O_2_ by RT-qPCR (Fig. 2*E* for *NQO1* and Fig. 2*F* for *OSGIN1*). Expression of *NQO1* increased with rising H_2_O_2_ levels, peaked at 40 µM H_2_O_2_ and declined at higher concentrations. By contrast, maximal *OSGIN1* expression coincided with the highest H_2_O_2_ concentration used, i.e., 80 µM H_2_O_2_. Overall, maximal NRF2 activation upon H_2_O_2_ exposure was stronger for *OSGIN1* (9.5-fold) than for *NQO1* (two-fold). For 160 µM H_2_O_2_, no usable mRNA amounts could be extracted to perform RT-qPCRs, due to the high cytotoxicity at this concentration. In summary, our cellular reporter shows superior sensitivity compared to similar systems (42) as substantial mCherry expression was already established at low, subtoxic H_2_O_2_ levels, and is therefore an ideal tool to disentangle how AnOs integrate in NRF2 signaling of cells.

### Surface Modification of AnOs with Cell-Penetrating Peptides Enhances Cellular Uptake.

**Fig. 3.**
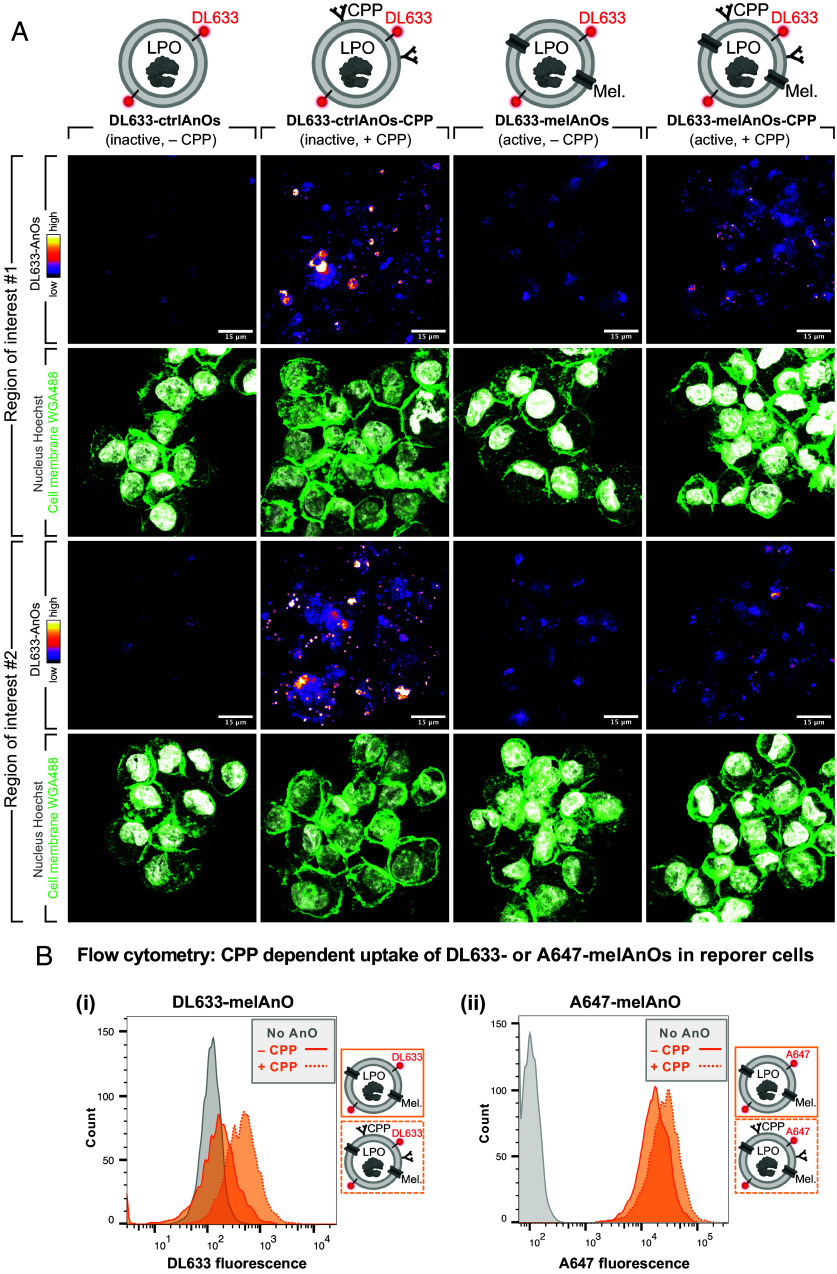
Uptake of AnOs in reporter cells is facilitated by surface functionalization with a dendritic cell–penetrating peptide. (*A*) Confocal microscopy. CPP-mediated uptake of DL633 labeled AnOs in reporter cells was monitored by confocal imaging 24 h after AnO administration. Two representative regions are shown for DL633-labeled ctrlAnOs (nonpermeabilized) and melAnOs (mel-permeabilized) either with or without CPP. Cell membranes were visualized by staining with AF488-labeled wheat-germ-agglutinin (WGA488; in green). Nuclei were stained with Hoechst (in gray scale). Images depict maximum intensity projections of z-stacks acquired by line-scanning confocal microscopy. Flow cytometry: Reporter cells were incubated with DL633-melAnOs or with A647-melAnOs with or without CPP as in (*A*). Cells were washed 3× by centrifugation and resuspension, and analyzed by flow cytometry for DL633 or A647 fluorescence to evaluate the fraction of cells that contained internalized fluo-melAnOs. (*B*) A total of N = 5,000 events per condition were analyzed, and histograms show (*i*) DL633 or (*ii*) A647 fluorescence intensities of live, single, GFP-positive gated cells.

To confer protection against H_2_O_2_ induced damages inside cells, where ROS is in direct contact with the intracellular machinery and thus most destructive, we aimed at improving the uptake of fluo-AnOs into cells. To validate that surface functionalization with CPPs indeed facilitated uptake, we incubated reporter cells for 24 h with DL633-labeled, active (mel-permeabilized) or inactive (nonpermeabilized) AnOs equipped with or without CPPs and examined them with microscopy (Fig. 3*A*). Cells were fixed with formaldehyde, and their borders visualized by staining the plasma membrane with AF488-labeled wheat-germ-agglutinin (WGA-488), and their nucleus by staining with Hoechst. Functionalization of DL633-ctrlAnOs with CPP greatly enhanced uptake into reporter cells as illustrated by a drastic increase in DL633 signal in two regions of interest (ROIs). Similarly, modification with CPPs boosted the uptake of DL633-melAnOs in cells, although the increase in DL633 fluorescence was slightly less pronounced than for DL633-ctrlAnOs. When comparing DL633-melAnOs to DL633-ctrlAnOs (both lacking CPPs), DL633-melAnOs showed a higher DL633 fluorescence. In order to explore whether a partial exposure of some charged residues of melittin at the AnOs’ hydrophilic interphase might influence the uptake into reporter cells independent of the CPP (43), we evaluated the mean fluorescence intensity of fluo-AnOs in adherent MCF7 breast cancer cells (*SI Appendix*, Fig. S4). Indeed, mel-permeabilized DL633- and A647-AnOs functionalized with CPP showed an enhanced uptake compared to the nonpermeabilized AnOs. Nevertheless, fluorescence images of reporter cells with internalized DL633-AnOs showed that surface functionalization of AnOs with CPPs made cellular uptake more efficient, and that the majority of DL633-AnOs were located throughout the cytoplasm. Moreover, internalization of DL633-AnOs without CPP was generally low which might be cell-type specific. Therefore, we also evaluated the CPP dependent uptake of active A647-labeled AnOs with or without CPP in MCF7 (*SI Appendix*, Fig. S5). We found that the uptake of mel-permeabilized AnOs was greatly facilitated by CPP also in adherent cells.

To evaluate the effect of CPP on AnO uptake in reporter cells more quantitatively, we assessed the internalization of DL633-melAnOs (with and without CPP) by measuring fluorescence intensities using flow cytometry (Fig. 3 *B, i*). After 24 h incubation with AnOs and repeated washings, quantification by flow cytometry revealed an increase in the median fluorescence intensity (MFI) of ~threefold (from ~160 to ~500) for DL633-melAnOs with CPP over those lacking CPP. This increase corroborates the beneficial effect of CPP on cellular uptake as already seen by fluorescence microscopy (Fig. 3*A*). Further, to generalize our results for the CPP-dependent uptake of AnOs into reporter cells, we treated reporter cells also with A647-labeled melAnOs with or without CPP. The treatment procedure was identical as for DL633-AnOs, and the A647 intensity in cells was quantified by flow cytometry as for DL633 (Fig. 3 *B, ii*). Again, CPP-functionalization boosted cellular uptake of permeabilized A647-melAnOs as seen by the increase in the MFI from ~1.5 × 10^4^ to ~2.5 × 10^4^. In summary, data from microscopy and cytometry experiments suggest a beneficial effect of CPP on the internalization of AnOs which is why we used only CPP-modified AnOs for further experiments.

### MelAnO-CPP Activity Dampens NRF2-Driven mCherry Expression and Modulates Endogenous NRF2 Target Genes.

**Fig. 4.**
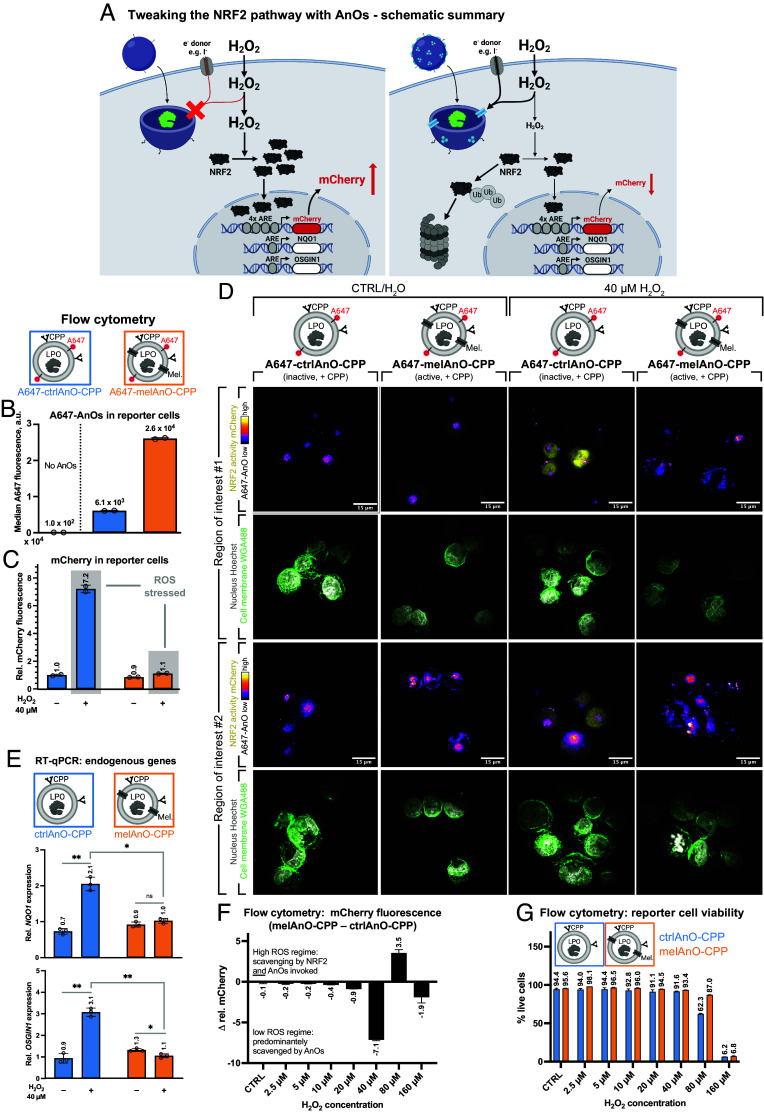
Scavenging of intracellular H_2_O_2_ by AnOs dampens NRF2-induced gene expression. Tweaking the NRF2 pathway with AnOs—schematic summary (*A*). *Left* panel: In reporter cells containing nonpermeable ctrlAnOs-CPP, excessive H_2_O_2_ levels cannot be detoxified and remain high, which consequently activates the NRF2-KEAP1 pathway. The high occupancy of AREs by NRF2 promotes mCherry expression. *Right* panel: Owing to their mel-permeabilized membrane, melAnOs-CPP are able to detoxify H_2_O_2_ inside cells which hinders nuclear translocation of NRF2, and thus, mCherry expression is reduced. Internalized AnOs dampen NRF2-driven mCherry expression by metabolizing H_2_O_2_ in reporter cells. A647-labeled AnOs (+CPPs) were supplemented to reporter cells, and cells were stimulated with 40 µM H_2_O_2_ for induction of NRF2. Uptake of AnOs (A647 fluorescence) and altered NRF2 activation state (mCherry fluorescence) were studied by flow cytometry (*B*: AnO uptake; *C*: NRF2 activation; duplicates with *N* = 5,000 events each; error bars = SD) and further by microscopy to correlate AnO uptake with reduced NRF2 activity (*D*). Cell membranes and nuclei were visualized as before. Images show maximum intensity projections of a stack from three z-layers for two representative ROIs for each condition. Effect of H_2_O_2_ scavenging by AnOs on endogenous NRF2 target genes. Nonlabeled AnOs (+CPPs) were added to reporter cells and cells were treated with 40 µM H_2_O_2_ as before. NRF2-driven transcription of endogenous targets *NQO1* and *OSGIN1* was determined by RT-qPCR in technical triplicates (*E*; error bars = SD). Data are representative for three independent experiments (one round is shown). Statistical significance was analyzed using a paired *t* test in Prism (ns *P* > 0.05, *P ≤ 0.05, **P ≤ 0.01, ***P ≤ 0.001). H_2_O_2_ scavenging by AnOs reduces NRF2-driven mCherry expression in a dose-dependent manner and increases cellular resilience against ROS. Cells were seeded as described and nonlabeled AnOs (+CPPs) were supplemented. NRF2 activation (mCherry fluorescence) and viability (violet LIVE/DEAD™ stain) were examined for increasing H_2_O_2_ concentrations with flow cytometry (duplicates with *N* = 5,000 events each; error bars = SD). (*F*) Differences in median mCherry fluorescence (Δ rel. mCherry = melAnO-CPP – ctrlAnO-CPP) and (*G*) total viability (in %) are shown.

To ask whether internalized AnOs can indeed modulate endogenous redox signaling events in cells, we exposed reporter cells to AnOs that were either enzymatically active (melAnOs-CPP) or inactive (ctrlAnOs-CPP) and then stimulated NRF2 activation by supplementing H_2_O_2_. In the case of ctrlAnOs-CPP, which lack melittin pores, electron donors and H_2_O_2_ have no access to the confined LPO (32), and thus, H_2_O_2_ levels are expected to remain high (Fig. 4 *A***,** *Left*). As a result, the NRF2-KEAP1 pathway is activated, nuclear NRF2 increases, and transcription of NRF2 target genes is induced, which is reported by increased mCherry. Contrarily, enzymatically active melAnOs-CPP should degrade intracellular H_2_O_2_ such that NRF2 is only moderately induced, and mCherry expression remains at basal levels (Fig. 4 *A*, *Right*). Similar expression patterns are expected also for endogenous target genes, *NQO1* and *OSGIN1*.

For testing the potential of melAnOs-CPP to reduce H_2_O_2_ levels inside cells, reporter cells were seeded and treated as outlined in *SI Appendix*, Fig. S6. In brief, 24 h after seeding, different types of A647-labeled AnOs (for uptake and simultaneous signaling studies) or nonlabeled AnOs (solely for signaling experiments) were added to the cells as indicated. After 7 h of incubation, potassium iodide (KI) was supplemented as electron donating cosubstrate, and 1 h later, H_2_O_2_ was added to cause oxidative stress. Finally, after 24 h, uptake of AnOs and stress-induced NRF2 activation was examined by flow cytometry, microscopy, and RT-qPCR (Fig. 4 *B–G*).

Reporter cells were treated with active or inactive A647-AnOs-CPP as outlined and uptake was verified under basal conditions (=no H_2_O_2_) by quantifying A647 fluorescence using flow cytometry (Fig. 4*B*). Fluorescence intensities for A647-ctrlAnOs-CPP (MFI = 6.1 × 10^3^) and A647-melAnOs-CPP (MFI = 2.6 × 10^4^) were higher than for cells without AnOs (MFI = 1.0 × 10^2^, background) indicating that both A647-AnO types were internalized.

Reporter cells harboring active A647-melAnOs-CPP or inactive A647-ctrlAnOs-CPP were then exposed to 40 µM H_2_O_2_, or to a corresponding amount of water (=basal conditions). In line with the H_2_O_2_-dependent NRF2 activation described before (Fig. 2*C*), mCherry fluorescence increased to 7.2-fold upon H_2_O_2_ in reporter cells containing inactive A647-ctrlAnOs-CPP (Fig. 4*C*). In contrast, the presence of permeabilized and therefore active A647-melAnOs-CPP reduced mCherry levels close to the basal conditions despite H_2_O_2_ exposure, suggesting that H_2_O_2_ was efficiently degraded by LPO catalysis in permeable A647-melAnOs-CPP, and thus NRF2 activation was significantly dampened.

To directly correlate intracellular A647-AnOs with the NRF2 activation state, we examined mCherry fluorescence in reporter cells under stressed und nonstressed conditions by confocal spinning disk microscopy (Fig. 4*D*). Clearly, control experiments in the absence of H_2_O_2_ stress (Fig. 4*D*, two left columns) show that mCherry expression is not induced through the treatment with either A647-ctrlAnOs-CPP or A647-melAnOs-CPP. In line with the findings from flow cytometry, most reporter cells that internalized inactive A647-ctrlAnOs-CPP expressed mCherry at high levels upon oxidative stress, while reporter cells containing A647-melAnOs-CPP expressed mCherry only at base levels as H_2_O_2_ was degraded and, thus, the NRF2 activation was dampened. Further, as H_2_O_2_ freely diffuses through cellular membranes, cells containing large numbers of active A647-melAnOs-CPP will reduce the overall H_2_O_2_ concentration for the entire cell population such that mCherry expression even remains low in cells with little or no AnO uptake.

To examine the effect of H_2_O_2_ detoxification on endogenous NRF2-regulated genes, we extracted mRNA from cells that contained nonfluorescent melAnOs-CPP or ctrlAnOs-CPP after H_2_O_2_ exposure and quantified the expression of *NQO1* and *OSGIN1* by RT-qPCR (Fig. 4*E*). We observed a significant increase in expression upon H_2_O_2_ exposure for both NRF2 target genes when inactive ctrlAnOs-CPP were present in cells, confirming the H_2_O_2_ induced transcriptional activation by NRF2. On the contrary, cells that harbored active melAnOs-CPP showed low expression of *NQO1* and *OSGIN1* when stressed by H_2_O_2_. These findings correspond to the trend observed for expression of the transgenic mCherry based NRF2 reporter at the protein level (Fig. 4 *C* and *D*) and mRNA level (*SI Appendix*, Fig. S7). Taken together, the data from RT-qPCR corroborate the trends from flow cytometry by providing further evidence that the NRF2-dampening effect by AnOs is not limited to the engineered reporter construct but also impacts endogenous gene regulation.

To ask how the AnO-mediated H_2_O_2_ scavenging integrates into the natural NRF2 antioxidant response, we tested whether there is a high-ROS stress regime where metabolization of H_2_O_2_ by melAnOs-CPP is exhausted such that the surplus H_2_O_2_ triggers activation of NRF2. For this, cells containing either nonfluorescent active melAnOs-CPP or inactive ctrlAnOs-CPP were exposed to increasing concentrations of H_2_O_2_ (without or with 2.5 to 160 µM H_2_O_2_) and the difference in mCherry fluorescence (=Δ rel. mCherry) from flow cytometry measurements was determined (Fig. 4*F* and *SI Appendix*, Fig. S8). H_2_O_2_ metabolization by active melAnOs-CPP is the predominant antioxidant mechanism up to 40 µM as indicated by a gradual decrease of Δ rel. mCherry fluorescence from −0.1 to −7.1 from basal conditions to 40 µM H_2_O_2_, respectively. At 80 µM H_2_O_2_, however, Δ rel. mCherry fluorescence turned positive, indicating that ROS scavenging invoked the NRF2 antioxidative response on top of the constantly active H_2_O_2_ degradation by melAnOs-CPP.

With regard to cell viability, as measured with the violet LIVE/DEAD stain in flow cytometry, the positive Δ rel. mCherry at 80 µM H_2_O_2_ correlates with a drop in cell viability for cells with ctrlAnOs-CPP (62.3% live) compared to cells containing melAnOs-CPP (87.0% live; Fig. 4*G*). This implies that cells with functional melAnOs-CPP were more resilient against ROS induced cell death by resorting to both AnO- and NRF2-based protection mechanisms, whereas cells with inactive ctrlAnOs-CPP were only protected by NRF2. At very high ROS levels, i.e., 160 µM H_2_O_2_, however, even the dual protection from AnOs and NRF2 was exceeded, and the majority of cells were dead; hence, mCherry levels were undetectable.

### Melittin-Permeabilized, Cell-Penetrating AnOs Increase Resilience against Oxidative Injury in Cells with Compromised ROS Defense.

**Fig. 5.**
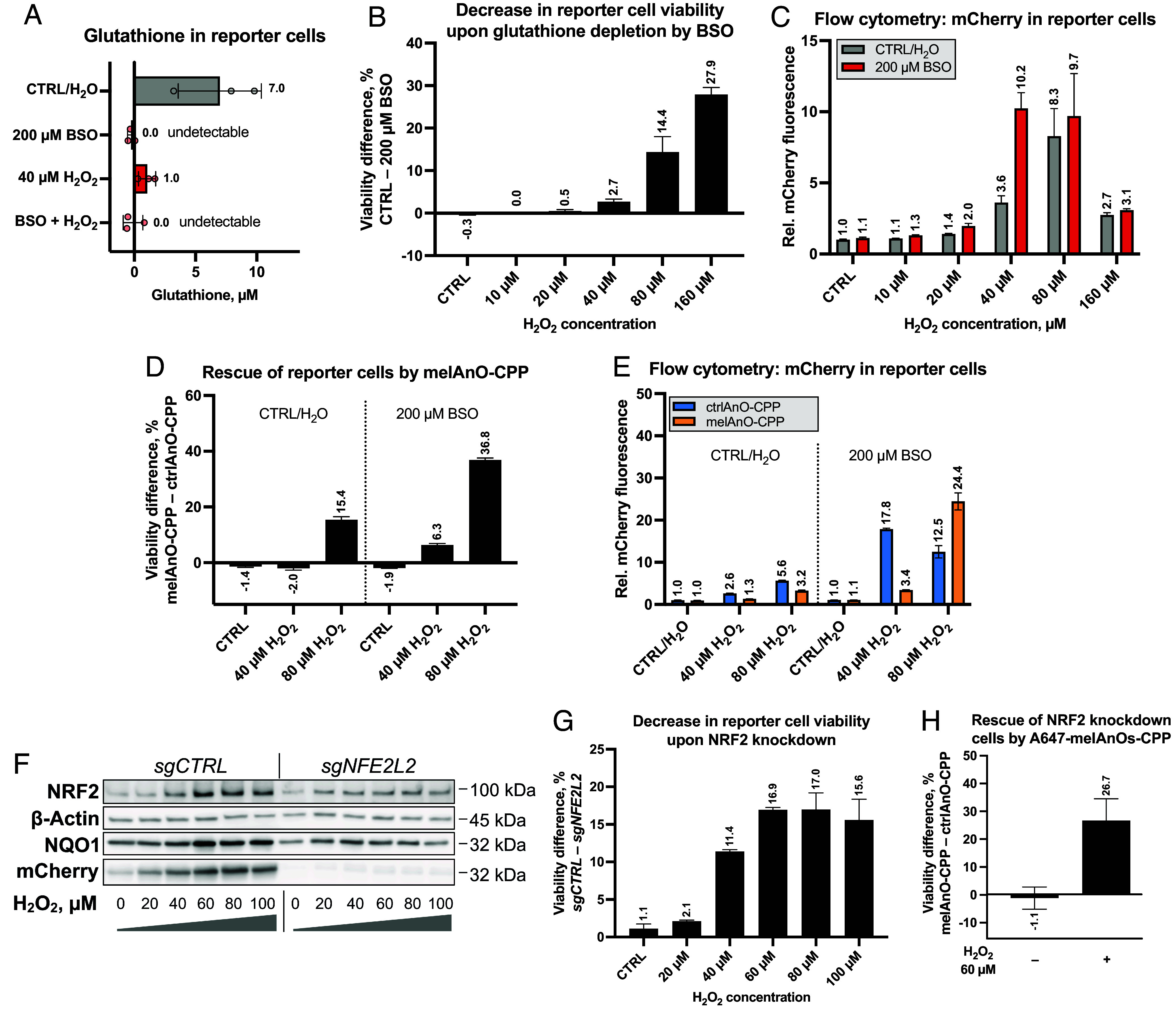
Internalized AnOs increase resilience against oxidative injury in those cells with compromised ROS defense. Treatment of reporter cells with the ROS-sensitizing drug buthionine sulfoximine (BSO) reduces cellular glutathione. Cells were seeded as described and treated with 200 µM BSO 6 h after seeding. Cells were further stimulated with or without 40 µM H_2_O_2_ as indicated. Glutathione was measured using a colorimetric assay (*A*; in technical triplicates; error bars = SD). Reducing glutathione, one of the main protective antioxidants in cells, increases susceptibility to oxidative cell death. Reporter cells were seeded and pretreated with 200 µM BSO and subjected to increasing levels of H_2_O_2_ (without or with 10 µM, 20 µM, 40 µM, 80 µM, and 160 µM H_2_O_2_). Cell viabilities of untreated vs. BSO-treated conditions (*B*: viability differences) as well as NRF2 activation (*C*) were determined by flow cytometry as described before (four replicates with *N* = 5,000 events each; error bars = SD). Internalized melAnOs-CPP confer protection to reporter cells with reduced glutathione. Reporter cells were seeded, pretreated with BSO as described and further supplemented with AnOs (+CPP). Cells were stressed using H_2_O_2_ (without or with 40 µM, 80 µM), and protection by functional melAnOs-CPP was determined by quantifying dead cells with flow cytometry (*D*). Viability data were correlated with NRF2 activation state by further quantifying mCherry amounts (*E*) in reporter cells (triplicates with *N* = 5,000 events each; error bars = SD). Knockdown of NRF2 causes reduced target gene expression and lower ROS defense. The gene of NRF2 (*NFE2L2*) was mutated in reporter cells by sgRNA-guided CRISPR-Cas9-induced DNA cleavage. Reporter cells transduced with a nontargeting sgRNA served as wild-type control (*sgCTRL*). (*F*) The knockdown was validated by subjecting whole cell lysates to immunoblotting and probing for total NRF2 and NRF2 target proteins (NQO1, mCherry) upon increasing levels of H_2_O_2_ (without or with 20 µM, 40 µM, 60 µM, 80 µM, and 100 µM H_2_O_2_). Reduced resilience against oxidative stress in the NRF2 knockdown was determined by flow cytometry as before (*G*: viability differences; triplicates with *N* = 5,000 events each; error bars = SD). A647-melAnOs-CPP confer protection against oxidative stress in reporter cells with depleted NRF2. Cells were seeded and supplemented with AnOs as described. Cells were stressed using 60 µM H_2_O_2_ and protection by functional A647-melAnOs-CPP was determined by quantifying dead cells with flow cytometry (*H*: viability differences; triplicates with *N* = 5,000 events each; error bars = SD).

Based on the finding that melAnOs-CPP assist NRF2 in the rescue of cells at high ROS regimes, we asked whether melAnOs-CPP can confer protection in situations where the endogenous ROS defense is compromised. As cellular glutathione, known to act as an important antioxidant in the body, is lowered by exposure to buthionine sulfoximine (BSO) (44), we either treated the reporter cells with 40 µM H_2_O_2_, which also decreased intracellular glutathione, or depleted cells entirely of glutathione with 200 µM BSO (Fig. 5*A*). When reporter cells were treated with 200 µM BSO 6 h after seeding and then further stressed with an increasing amount of H_2_O_2_ (0 to 160 µM) as described before (*SI Appendix*, Fig. S6), we observed an increased susceptibility to oxidative cell death (Fig. 5*B* and *SI Appendix*, Fig. S9). Accordingly, mCherry in reporter cells that received the BSO pretreatment peaked at lower H_2_O_2_ concentrations (i.e., 40 µM) than in cells that did not receive BSO (i.e., 80 µM) (Fig. 5*C*). This might be due to the lowered glutathione-based defense in cells with BSO pretreatment which renders them more sensitive to ROS as reflected by earlier NRF2 activation and apoptosis at lower H_2_O_2_ levels. At 160 µM H_2_O_2_, the mCherry fluorescence drops due to the low cell viability for both conditions. Internalization of active melAnOs-CPP can complement the missing glutathione defense and confers protection against cell death at 40 µM and 80 µM H_2_O_2_ as seen by a viability increase of 6.3% for 40 µM H_2_O_2_ and 36.8% for 80 µM H_2_O_2_ in comparison to cells harboring inactive ctrlAnOs-CPP, and thus confirms the protecting properties of internalized active AnOs (Fig. 5*D*). Accordingly, at a low ROS regime (= 40 µM H_2_O_2_), melAnOs-CPP provided the dominant ROS defense which sidesteps NRF2-driven mCherry induction in both BSO pretreated and untreated cells, with generally higher NRF2 activation levels in BSO sensitized cells (Fig. 5*E*). At high ROS stress (=80 µM H_2_O_2_) and missing glutathione defense, however, melAnOs-CPP based protection had to be complemented by robustly activated NRF2, which explains the high mCherry levels (~ 24-fold) at this condition, while cells with inactive ctrlAnOs-CPP showed lower mCherry amounts due to higher apoptosis rates as described before (Fig. 5*D*).

To further investigate the effect of AnOs-based protection in situations with lowered ROS defense, we knocked down NRF2. The gene of NRF2 (*NFE2L2*) was mutated in reporter cells by sgRNA-guided CRISPR-Cas9-induced DNA cleavage (45, 46). Reporter cells transduced with a nontargeting sgRNA expressed wild-type NRF2 and hence served as control (*sgCTRL*). Knockdown of NRF2 caused reduced target gene expression and lower ROS defense. The knockdown was validated by subjecting whole cell lysates to immunoblotting and probing for total NRF2 and NRF2-regulated proteins (NQO1, mCherry) upon increasing levels of H_2_O_2_ (Fig. 5*F*). Upon increasing oxidative stress, NRF2 stabilized less in NRF2 knockdown cells concomitant with decreased target protein expression seen by reduced NQO1 and basically entirely depleted mCherry protein. Viability analysis by flow cytometry showed that depletion of NRF2 reduced the resilience of reporter cells against oxidative stress drastically starting from 60 µM H_2_O_2_ onward (Fig. 5*G* and *SI Appendix*, Fig. S10). To assess whether active AnOs could counteract the effect of NRF2 knockdown on cell viability, cells containing active or inactive AnOs with an A647 label (i.e., A647-melAnOs-CPP or A647-ctrlAnOs-CPP) were stressed with 60 µM H_2_O_2_ (Fig. 5*H* and *SI Appendix*, Fig. S11). Notably, A647-melAnOs-CPP rescued NRF2 knockdown cells from oxidative stress, as seen by an increase in viability of 26.7 %, while there was no difference between cells containing active or inactive AnOs under basal conditions (= no H_2_O_2_).

In summary, our readouts show that AnOs can even tailor redox signaling when cells were sensitized to ROS by BSO-mediated glutathione depletion, and, at higher ROS levels, protect such sensitized cells from apoptosis. Moreover, cell death by sensitization though knockdown of the antioxidant master regulator NRF2 could be also alleviated by AnO supplementation.

### CPP Also Facilitates Uptake of A647-AnOs in Human Lymphocytes Where Active A647-melAnOs-CPP Confer Protection against Oxidative Injury.

**Fig. 6.**
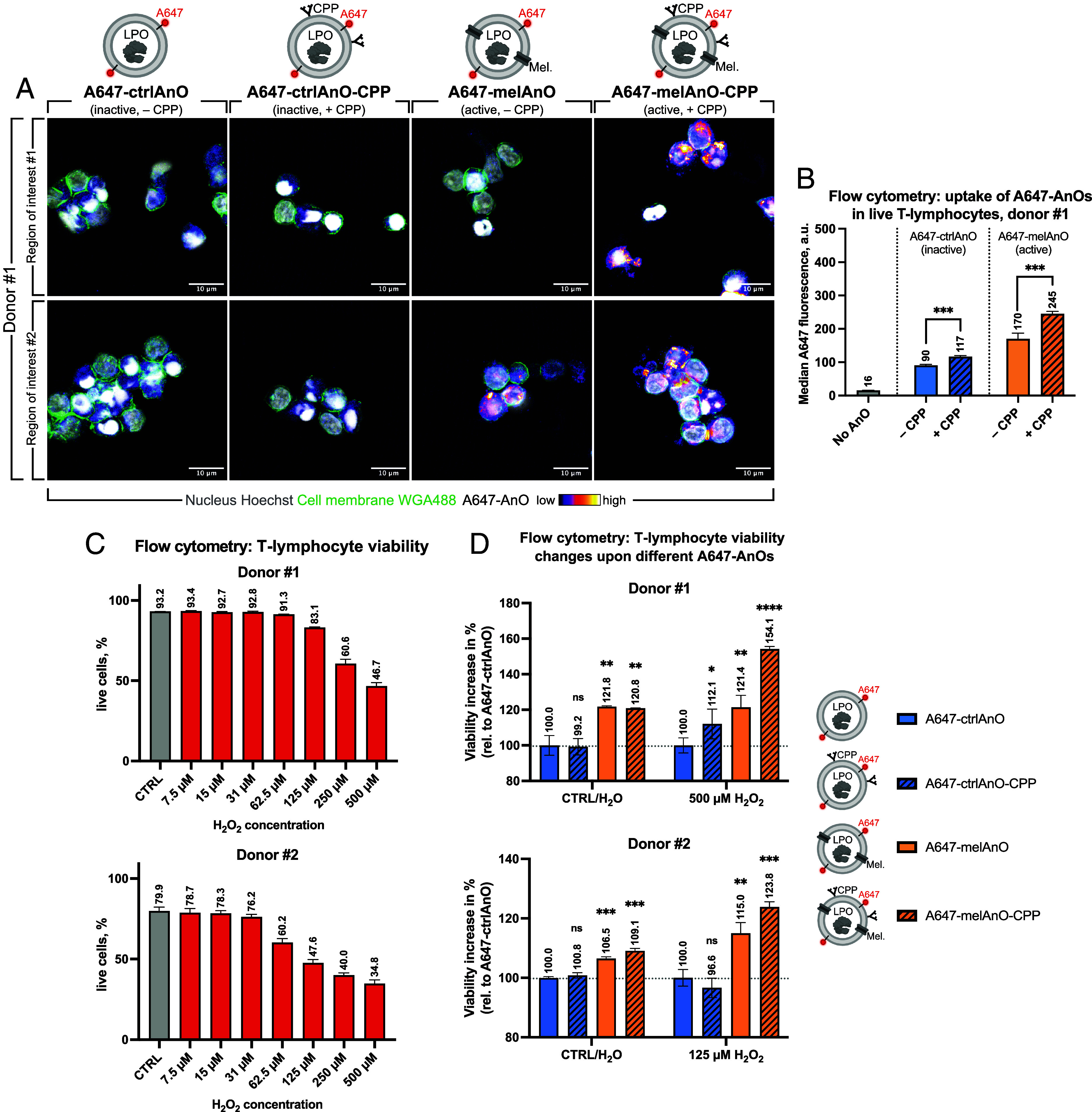
Verification that human primary cells can be protected against oxidative injury using human peripheral blood mononuclear cells (PBMCs). Uptake of A647-AnOs in PBMCs is enhanced by CPP. Peripheral blood mononuclear cells (PBMCs; donor #1) were seeded at a density of 100,000 in 100 µL per well and AnOs were added after 24 h. A647-labeled ctrlAnOs or melAnOs with or without CPPs were supplemented as indicated. CPP-enhanced uptake of A647-labeled AnOs was monitored by Airyscan superresolution microscopy 24 h after AnO administration (*A*). Two representative regions are shown for each condition. Cell membranes were visualized by staining with AF488-labeled wheat-germ-agglutinin (WGA488; in green). Nuclei were stained with Hoechst (in gray scale). Images depict maximum intensity projections of z-stacks. CPP-facilitated uptake of A647-AnOs in PBMCs (donor #1) was also quantified by flow cytometry with a focus on the T lymphocyte population (*B*; four replicates with *N* = 5,000 events each). Data were tested for statistical significance in Prism using a paired t test (ns P > 0.05, *P ≤ 0.05, **P ≤ 0.01, ***P ≤ 0.001, ****P ≤ 0.0001) and normal distribution was visually confirmed by QQ-plots. T lymphocytes were identified for further analyses by size and granularity in FSC:SSC plots as determined by antibody-based staining against CD3 (T lymphocytes), CD14 (monocytes), and CD19 (B lymphocytes) for distinguishing them from other PBMC types (*SI Appendix*, Fig. S12). Decreasing T lymphocyte viability upon increased oxidative stress. PBMCs (donor #1 and #2) were seeded as described and stressed with increasing levels of H_2_O_2_ (0 to 500 µM). Viabilities of the T lymphocyte population were determined with violet LIVE/DEAD™ stain by flow cytometry (*C*; triplicates with *N* = 5,000 events each; error bars = SD). Active A647-AnOs confer protection against oxidative stress in human T lymphocytes. PBMCs (donor #1 and #2) were seeded and supplemented with AnOs as described. Cosubstrate potassium iodide was added 1 h prior to stressing cells of donor #1 or #2 with 500 or 125 µM H_2_O_2_, respectively, and protection by functional A647-melAnOs-CPP was determined by quantifying changes in T lymphocyte viability with flow cytometry (*D*). Relative viabilities (normalized to condition with A647-ctrlAnO = 100 %) were calculated for each condition (four replicates with *N* = 5,000 events each). AnO-mediated protection was tested for statistical significance in Prism using a paired *t* test (with respect to the ctrlAnO condition; ns *P* > 0.05, *P ≤ 0.05, **P ≤ 0.01, ***P ≤ 0.001, ****P ≤ 0.0001) and normal distribution was visually confirmed by QQ-plots.

To verify whether our results can be generalized, we repeated key experiments using other cell types, i.e., with primary peripheral blood mononuclear cells (PBMCs) from healthy human donors. The beneficial effect of CPP surface modification on cellular uptake seen in K562 reporter cells could be validated for PBMCs (donor #1) with a special focus on the T lymphocyte population. Airyscan superresolution microscopy revealed an advantageous effect of CPP on the uptake especially for A647-melAnOs-CPP over A647-melAnOs (Fig. 6*A*). This was further confirmed by flow cytometry experiments, where T lymphocytes showed significantly elevated A647 fluorescence for CPP-functionalized AnOs for both A674-melAnOs-CPP over A674-melAnOs or A674-ctrlAnOs-CPP over A674-ctrlAnOs (Fig. 6*B*). The T lymphocyte subpopulation was demarcated from other PBMC types using a stringent gate in FSC-A:SSC-A plots, which was determined in advance by staining with labeled antibodies against CD3 (T lymphocytes), CD14 (monocytes), and CD19 (B lymphocytes) (*SI Appendix*, Fig. S12). Interestingly, for donor #1, only 0.1% of all events (excluding debris) were CD19^+^, while 64.6% were CD3^+^ and 3.9% were CD14^+^, while for donor #2, 0.6% were CD19^+^, 74.2% were CD3^+^ and 4.2% were CD14^+^ (*SI Appendix*, Fig. S12). Viabilities of T lymphocytes from donor #1 and #2 gradually decreased upon increasing H_2_O_2_ concentrations (0 to 500 µM) as determined by violet LIVE/DEAD stain with flow cytometry. Donor #1 showed a higher resilience against ROS with 46.7% live cells at 500 µM H_2_O_2_, whereas donor #2 had a similar viability of 47.6% at only 125 µM H_2_O_2_ (Fig. 6*C*). Finally, we checked whether AnOs can increase protection against ROS by supplying them to PBMCs and then stressing donor #1 with 500 µM and donor #2 with 125 µM H_2_O_2_, respectively. T lymphocyte viabilities for both donors #1 and #2 were the highest when A647-melAnOs-CPP had been supplemented (Fig. 6*D*), which increased the viability to 154.1% for donor #1 and 123.8% for donor #2 when comparing to the condition with inactive A647-ctrlAnO that lacked CPP (=100 %). Taken together, these results confirm the ROS-protecting properties of functional AnOs, as their protective effect can also be seen in primary blood cells. AnOs can boost cellular defense most efficiently when internalization is enhanced by CPP on their surface.

## Discussion and Conclusion

As excessive cellular ROS levels are associated with aging and with several chronic inflammatory diseases (5–7), we addressed whether the redox sensitive NRF2 pathway can be tweaked in cells by carefully designed, ROS-degrading artificial nano-organelles. This was accomplished by engineering an advanced generation of artificial nano-organelles with dual modality: one geared toward reverting elevated ROS signaling back to homeostasis inside cells, where ROS are most damaging, and the other toward tracking and visualizing the artificial organelles within the cells. In view of future medical applications, the AnOs were designed with a nonbiodegradable PMOXA surface to improve systemic circulation time and decrease immunogenicity. In order to enable enzymes encapsulated inside AnOs to detoxify ROS in situ, the membrane was permeabilized by insertion of melittin pores in controlled conditions to preserve the integrity of the AnOs, while the simultaneous insertion of fluorescent dyes supported their precise localization inside cells. In addition, using a mixture of diblock copolymers provided the necessary flexibility and stability of the AnOs while supporting their functionalization with dendritic cell–penetrating peptides for cell uptake. Such AnOs with improved properties are expected to prolong their retention times to assist cells with ROS detoxification in pathologies that arise from ROS imbalance. The cell-penetrating peptides exposed on the surface of AnOs efficiently promoted their uptake in leukemic K562 cells and in normal T lymphocytes.

To unravel contributions of the KEAP1-NRF2 pathway in balancing cellular ROS levels, a reporter human leukemic cell line was created that expresses the fluorescent mCherry protein in response to increased intracellular H_2_O_2_. The H_2_O_2_-degrading activity of internalized AnOs reduced NRF2 transcriptional activity in these cells, a downregulation which indicates that oxidative stress can indeed be alleviated by the help of functional AnOs. Downregulation of endogenous NRF2 target genes provided further evidence of the influence of our AnOs on ROS metabolism.

Our data show that functional AnOs can substitute the NRF2 pathway in ROS scavenging at the low ROS regime. At the high ROS regime, where the endogenous NRF2-activated defense is exhausted, AnOs successfully complemented the cell’s existing antioxidant enzyme repertoire. Interestingly, when endogenous ROS protection mechanisms had been compromised, either by knockdown of NRF2 or glutathione depletion, intracellular AnOs substituted the endogenous ROS defense effectively and conferred protection against oxidative stress-induced apoptosis. Moreover, to support the therapeutic premise of these AnOs-CPP, we repeated key experiments with peripheral blood mononuclear cells from healthy human donors. Importantly, the CPP-mediated uptake of AnOs by these immune cells also provided protection from ROS-induced cell death.

While H_2_O_2_ metabolization was demonstrated here for the enzyme LPO, other H_2_O_2_-degrading enzymes can be envisioned, too. For example, H_2_O_2_ degradation by catalase and thus independent of available electron donors might even be advantageous for potential in vivo applications. Further, AnOs that target harmful superoxide radicals (O_2_^−^), which arise from imperfect electron transport in cells with high mitochondrial activity, could be generated by encapsulation of O_2_^−^ metabolizing enzyme superoxide dismutase. The resulting H_2_O_2_ could then be degraded by LPO-containing AnOs in a cascade reaction. Such tandem of ROS-degrading AnOs, enhancing existing reaction cascades or even carrying out cascade reactions themselves, not only have the potential to significantly advance the therapy of elevated cellular ROS, but also to contribute more broadly to the repair of disturbed cell functions associated with progressive diseases, including fibrosis, arthritis, neurodegenerative diseases, and many others. Our ROS scavenging artificial organelles might also be beneficial for a variety of applications in bioprocesses for improved recombinant protein expression. Large-scale biopharmaceutical production of biologics depends on the overexpression of heterologous proteins by optimized cells, such as Chinese hamster ovary cells. High‐level expression of foreign proteins requires metabolic adaptions including increased energy production and improved secretory capacity, which, in turn, can lead to a rise of ROS generated through leaks in the cellular respiratory chain. Utilizing nanotechnology approaches for expanding on the cellular ROS defense by introducing ROS scavenging artificial organelles into such production cells might thereby help to alleviate oxidative stress, as for example generated during fermentation processes, for improved cell growth, productivity, and reduced product heterogeneity.

## Materials and Methods

See *SI Appendix*, *Materials and Methods* for technical details for each method.

### Preparation of CPP-Functionalized Fluo-AnOs.

AnOs were prepared as recently described (32), except that thin polymer films were dried together with membrane dyes (DL633 or A647) followed by rehydration in PBS.

### AnO Characterization by Static (SLS) and Dynamic Light Scattering (DLS).

Multiangle light scattering data were recorded on a spectrometer (LS Instruments). Scattering angles between 40 and 135° were recorded. Hydrodynamic radii (*R*_h_) and polydispersity indices (PDI) were obtained from DLS measurements. Radius (*R*) and radius of gyration (*R_g_*) were derived from SLS analyses.

### Transmission Electron Microscopy (TEM).

In preparation for TEM, aliquots of AnOs in PBS were deposited on a freshly glow-discharged, carbon-coated, parlodion-(2% in n-butyl acetate) copper grid. TEM images were recorded on a CM100 transmission electron microscope (Philips).

### Nanoparticle Tracking Analysis (NTA).

Size (hydrodynamic radius, R_h_) and concentration (particles/mL) of AnOs were analyzed by NTA using a NanoSight NS300 (Malvern Panalytical Ltd.) instrument. Samples were freshly diluted at 1:1,000 in filtered PBS and applied to the integrated flow cell at a flow rate of 100 μL min^−1^.

### Fluorescence Correlation Spectroscopy (FCS).

FCS experiments were performed on a Zeiss LSM 880 laser-scanning microscope. Autocorrelation curves were fitted by a two-component model as previously described (24).

### Lactoperoxidase (LPO) Enzymatic Assay.

LPO activity was assayed as previously described (24).

### Culturing of Cells.

For details about maintaining cultures of K562 reporter cells, HEK293T cells for virus production and primary peripheral blood mononuclear cells (PBMCs) see *SI Appendix*.

### Lentiviral Infections.

K562 cells were lentivirally modified to express mCherry under ARE control and EGFP under CMV promotor control. Moreover, NRF2 was knocked down with CRISPR-Cas9 by cloning guide RNA sequences (NRF2/*sgNFE2L2* = GGACATTGAGCAAGTTTGGG; nontargeting control *sgCTRL* = GGCCTGCCCTAAACCCCGGA) into the plenti-CRISRP-v2 vector (#52961, Addgene) as described in previous studies (45, 46).

### NRF2 Activity Assay for K562 Reporter Cells Using Flow Cytometry.

Cells were seeded in PRMI medium and, if applicable, pretreated with BSO 6 h after seeding. Then, 24 h post seeding, AnOs were added. After 7 h, potassium iodide as electron donor was added, followed by H_2_O_2_ after an additional 1 h. Then, 24 h after this, cells were processed for flow cytometry, microscopy, or RT-qPCR to study NRF2 activation, viability, or AnO internalization.

In preparation for flow cytometry, noninternalized AnOs were washed away by repeated centrifugation and resuspension steps, and dead cells were labeled with violet live/dead™ stain (#L34963, Thermo Scientific) before fixation with 4% formaldehyde. Fluorescence levels of mCherry, GFP, fluo-AnOs, and viability dye in reporter cells were quantified on a BD LSRFortessa cytometer.

### Killing and Rescue Assay with PBMCs Using Flow Cytometry.

Collection of PBMCs and conducted experiments were approved by the Kantonale Ethikkommission Zürich (KEK-ZH-Nr. 2012-0111), and written consent was obtained from all subjects. See *SI Appendix* for details about PBMC isolation from buffy coats and culturing. PBMCs were seeded 24 h prior to treatments. AnO supplementation, as well as treatment with potassium iodide and H_2_O_2_ were identical as for K562 reporter cells, but in PBMC medium. After an incubation of 24 h with H_2_O_2_, PBMCs were processed for flow cytometry (BD LSRFortessa) and/or microscopy (Zeiss LSM 880 AxioObserver).

PBMCs were prepared for flow cytometry as K562 reporter cells. Moreover, to determine gates for the live and dead T lymphocyte population in FSC-A:SSC-A plots, PBMCs were stained also for CD3 (T lymphocytes), CD14 (monocytes), and CD19 (B lymphocytes).

ROS protection by AnOs on immune cells was assessed by quantifying dead cells after staining with violet live/dead™ stain followed by fixation with 4% formaldehyde as for K562 cells; the antibody-based staining was not repeated each time. AnO uptake and protection against ROS was only determined for the T lymphocyte population as identified by lineage marker staining.

### Real-Time Quantitative PCR (RT-qPCR) and Western Blotting.

Quantification of NRF2 activation (and/or NRF2 knockdown efficiency) on protein and mRNA levels was done by western blotting and RT-qPCR using standard protocols.

### Quantification of Glutathione.

Glutathione was quantified via a photometric assay (#703002, Cayman Chemical) according to the manufacturer’s protocol.

### Fluorescence Microscopy.

CPP facilitated uptake of AnOs in K562 reporter cells (Fig. 3) was investigated on a Leica TCS SP8 line scanning microscope. For correlating AnO uptake with NRF2 controlled mCherry expression under oxidative stress (Fig. 4), K562 reporter cells were imaged on a Nipkow spinning disk confocal microscope. CPP-facilitated uptake of AnOs in PBMCs (Fig. 6) was imaged on a Zeiss LSM 880 AxioObserver in Airyscan superresolution mode.

## Supplementary Material

Appendix 01 (PDF)

## Data Availability

Raw data and processed data from all main and *SI Appendix* figures in digital format data have been deposited in ETH Zurich Research Collection (https://doi.org/10.3929/ethz-b-000669761) (47). All other data are included in the manuscript and/or *SI Appendix*.
