# Supplementary material for: Tweaking the NRF2 signaling cascade in human myelogenous leukemia cells by artificial nano-organelles
Source: Proc Natl Acad Sci U S A. 2024 May 22;121(22):e2219470121. doi: 10.1073/pnas.2219470121 (PMC11145192; doi:10.1073/pnas.2219470121)
Supplement: Supplementary file 1 — Appendix 01 (PDF) [file pnas.2219470121.sapp.pdf]

PNAS

Supplementary Information

Tweaking the NRF2 signaling cascade in human myelogenous leukemia cells by artificial nano-organelles

Konstantin M. P. Wolf[1,a,c], Viviana Maffeis[1,b,c], Cora-Ann Schoenenberger[b,c], Tamara Zünd[a], Liron Bar-Peled[d], Cornelia G. Palivan*[,b,c], Viola Vogel*[,a,c]

([1]Equal contributors, *co-corresponding authors)

[a]Laboratory of Applied Mechanobiology, Institute of Translational Medicine, Department of Health Sciences and Technology, ETH Zurich, Gloriastrasse 37/39, 8092 Zurich, Switzerland

[b]Department of Chemistry, University of Basel, Mattenstrasse 24a, BPR1096, 4002 Basel, Switzerland

[c]NCCR-Molecular Systems Engineering, 4002 Basel, Switzerland

[d]Center for Cancer Research, Massachusetts General Hospital/Harvard Medical School, MA 02129 Boston, USA

Contact:

Prof. Dr. Viola Vogel: viola.vogel@hest.ethz.ch, +41 44 632 08 87, ETH Zurich, Gloriastrasse 37/39, 8092 Zürich, Switzerland

Prof. Dr. Cornelia Palivan: cornelia.palivan@unibas.ch, +41 61 207 38 39, University of Basel, Mattenstrasse 24a, BPR1096, 4002 Basel, Switzerland

This file includes:

Figures S1-S12:

Figure S1. AnOs with surface coupled CPP.
Figure S2. Normalized FCS autocorrelation curves of A647-ctrlAnO-CPP.
Figure S3. LPO functionality of active and inactive A647-AnOs with or without CPP.
Figure S4. Uptake of inactive and active CPP-functionalized DL633-AnOs and A647-AnOs in MCF-7 cells.
Figure S5. CPP-dependent uptake of active A647-AnOs in MCF-7 cells.
Figure S6. Experimental timeline of treating reporter cells with AnOs and required substrates.
Figure S7. Dampened NRF2 transcriptional activation by scavenging intracellular H_2O_2 with AnOs (RT-qPCR of mCherry).
Figure S8. H_2O_2 scavenging by AnOs reduces NRF2 driven mCherry expression in a dose

dependent manner (rel. mCherry levels).

Figure S9. Reduced glutathione increases susceptibility to oxidative cell death (cell viabilities).

Figure S10. Knockdown of NRF2 lowers cellular ROS defense (cell viabilities).

Figure S11. A647-melAnOs-CPP confer protection against oxidative stress in reporter cells with depleted NRF2 (cell viabilities).

Figure S12. Identification of immune cell subpopulations in PBMCs under basal (= no H_2O_2) and stressed conditions (= 500 µM H_2O_2) using flow cytometry.

and Tables S1-S3:

Table S1. Quantification of dyes per fluo-AnOs by FCS.

Table S2. Brightness measurements of DyLight 633 (DL633) or Atto647N DOPE (A647) labelled fluo-AnOs as obtained by FCS.

Table S3. Particle concentration of A647-AnOs by nanoparticle tracking analysis (NTA).

Supplementary Materials & Methods

A

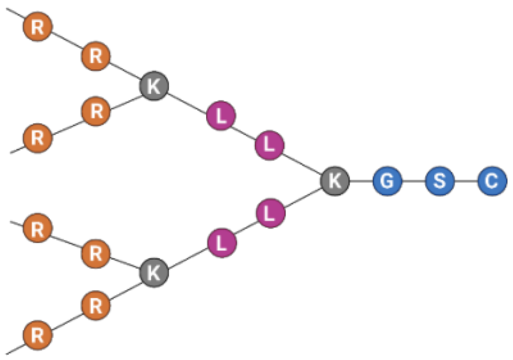

Cell penetrating peptide (CPP)

CPP: $(RR)_4(KLL)_2KGKC^{**}$

**= DBCO = Dibenzocyclooctyne-maleimide

Chemical Formula: $C_{126}H_{220}N_{50}O_{22}S$

Exact Mass: 2817.74

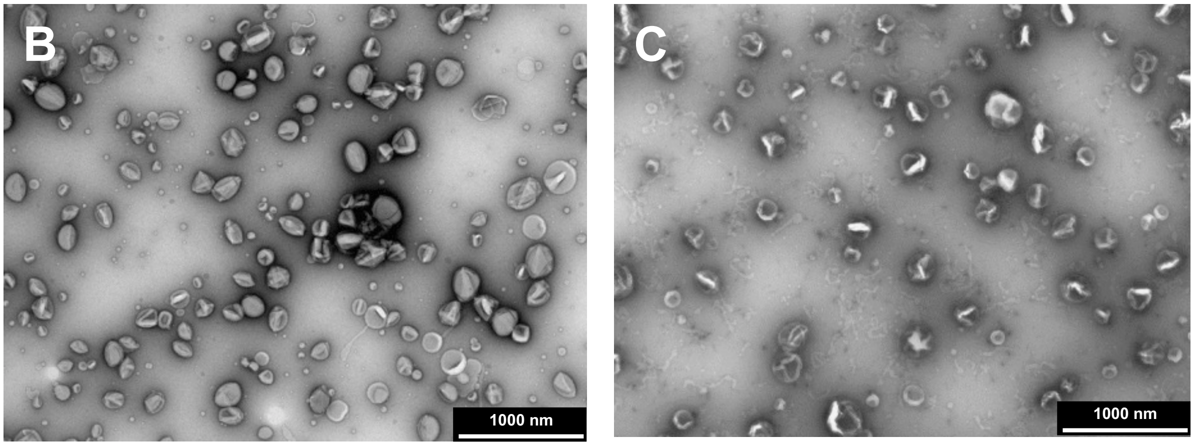

Figure S1. AnOs with surface coupled CPP. (A) Chemical composition of the DBCO-functionalized cell penetrating peptide (CPP) used for coupling to the surface of AnOs. One-letter code identifies amino acids constituting the dendrimer. **(B)** TEM micrographs of DL633-melAnOs-CPP (active), harboring fluorescent dyes in their membrane. **(C)** TEM micrographs of A647-melAnOs-CPP (active) showing the typical morphology of collapsed polymersomes. Scale bar: 1000 nm.

Table S1. Quantification of dyes per fluo-AnOs by FCS.

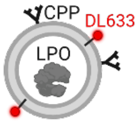

**FCS (DL633-ctrlAnOs-CPP)**
**Diffusion time (μs)**
τ (dylight-633): 53
τ (AnOs): 4231
dye/AnOs: 88 ± 10

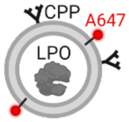

**FCS (A647-ctrlAnOs-CPP)**
**Diffusion time (μs)**
τ (Atto 647N DOPE): 50
τ (AnOs): 4812
dye/AnOs: 274 ± 8

Table S2. Brightness measurements of DyLight 633 (DL633) or Atto647N DOPE (A647) labelled fluo-AnOs as obtained by FCS.

**Counts per molecule**	
**DyLight 633 NHS ester**	4.624
DL633-melAnO-CPP	399.21
DL633-ctrlAnO-CPP	406.64
DL633-melAnO	382.81
DL633-ctrlAnO	388.75

**Counts per molecule**	
**Atto647N DOPE**	1.344
A647-melAnO-CPP	369.34
A647-ctrlAnO-CPP	368.05
A647-melAnO	356.43
A647-ctrlAnO	354.06

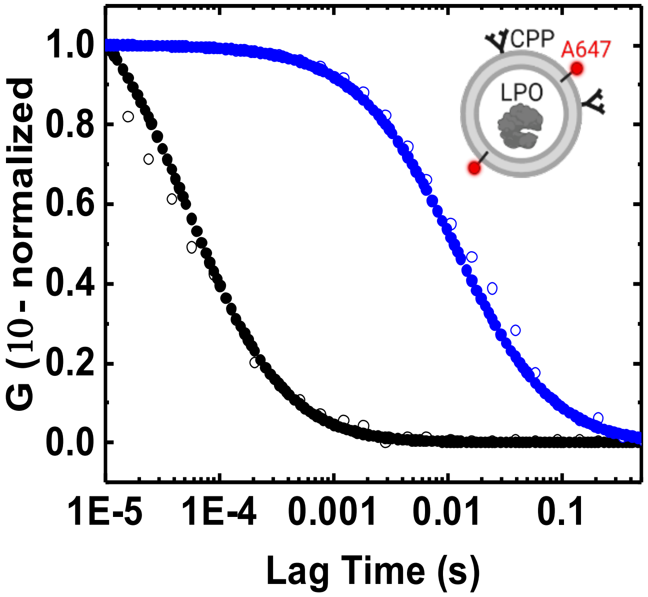

Figure S2. Normalized FCS autocorrelation curves of A647-ctrlAnO-CPP. Normalized FCS autocorrelation curves (open circles: raw data; filled circles: fitted curves) of free Atto 647N DOPE dye (black) and Atto 647N DOPE-labeled A647-ctrlAnOs-CPP (blue).

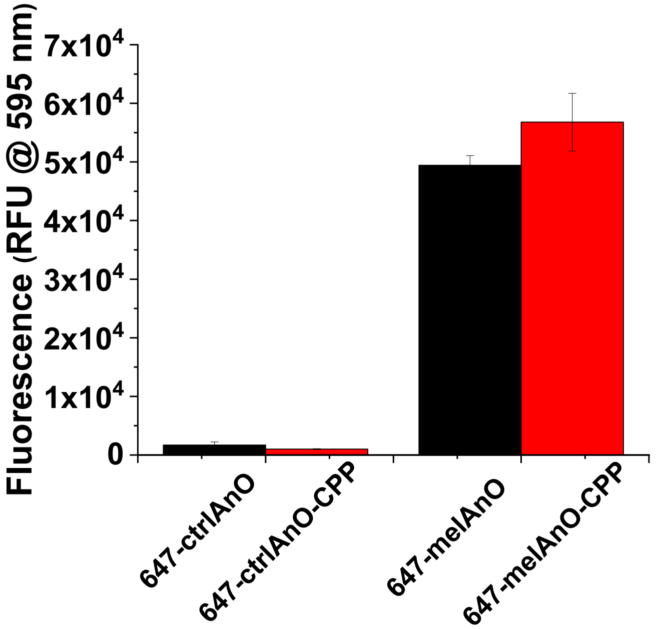

Figure S3. LPO functionality of active and inactive A647-AnOs with or without CPP. Enzymatic activity was determined by monitoring the conversion of AmplexRed to a resorufin-like product, RLP, at 560 nm after 20 min.

Table S3. Particle concentration of DL633-AnOs and A647-AnOs by nanoparticle tracking analysis (NTA).

**NTA**	
	**Particle concentration (particles/mL)**
DL633-melAnO-CPP	$1.23 * 10^{12}$
DL633-ctrlAnO-CPP	$1.35 * 10^{12}$
DL633-melAnO	$1.13 * 10^{12}$
DL633-ctrlAnO	$1.12 * 10^{12}$

**NTA**	
	**Particle concentration (particles/mL)**
A647-melAnO-CPP	$1.45 * 10^{12}$
A647-ctrlAnO-CPP	$1.41 * 10^{12}$
A647-melAnO	$1.30 * 10^{12}$
A647-ctrlAnO	$1.35 * 10^{12}$

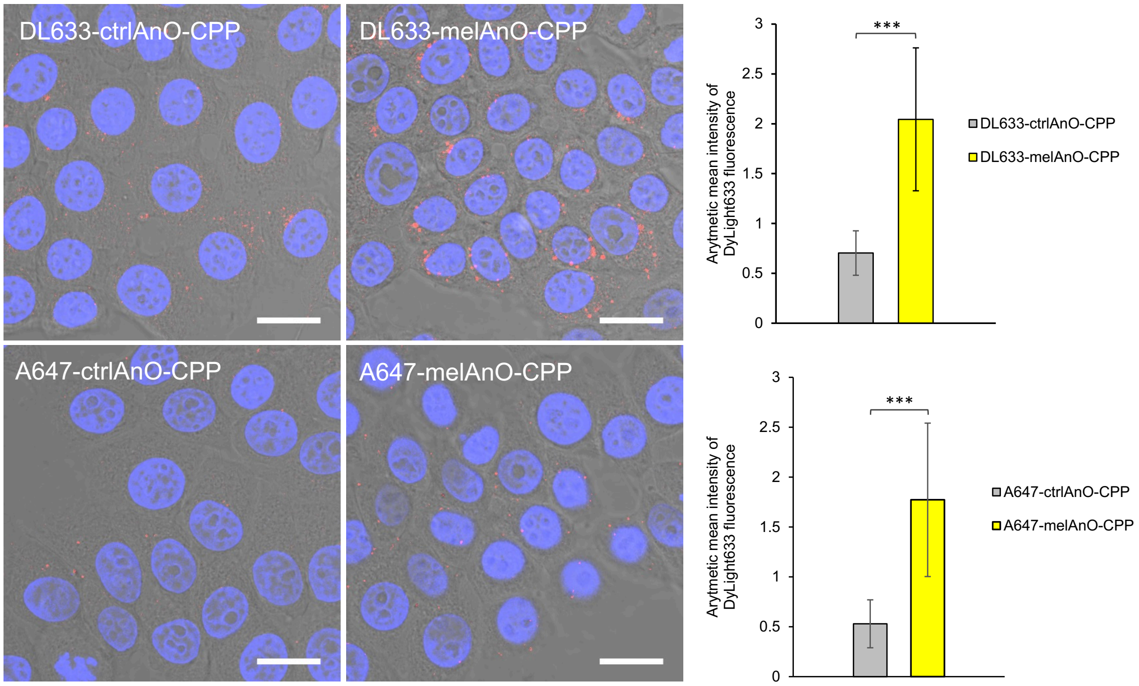

Figure S4. Uptake of inactive and active CPP-functionalized DL633-AnOs and A647-AnOs in MCF-7 cells. Images of MCF-7 cells incubated with different samples of DL633-AnOs or A647-AnOs for 24 h obtained by confocal laser scanning microscopy. Cells were fixed with 4 % paraformaldehyde and cell nuclei were stained with Hoechst 33342. Images present transmission light channel merged with blue fluorescence of cell nuclei (Hoechst 33342) and red fluorescence of DL633-AnOs (top panels) and A647-AnOs (bottom panels). Scale bar = 20 µm.

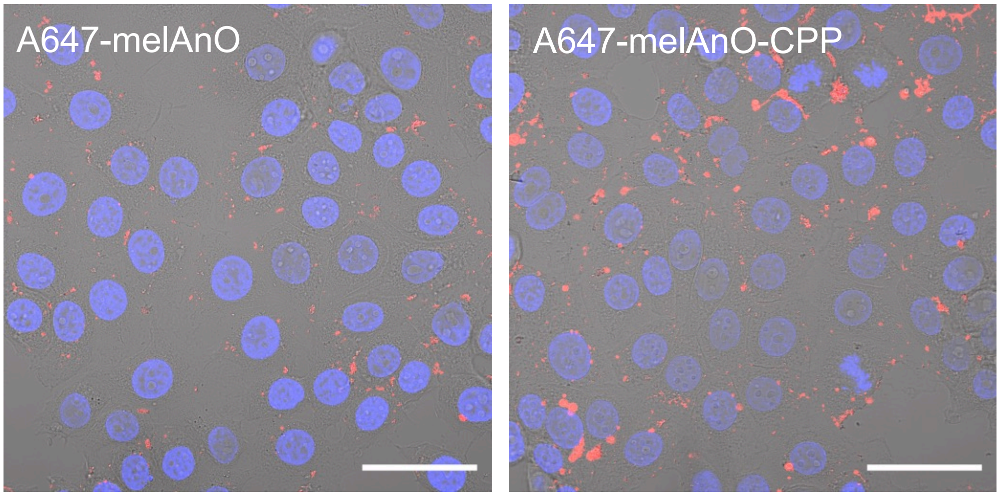

Figure S5. CPP-dependent uptake of active A647-AnOs in MCF-7 cells. Images of MCF-7 cells incubated with different samples of A647-AnOs for 24 h as obtained by confocal laser scanning microscopy. Cells were fixed with 4 % paraformaldehyde and cell nuclei were stained with Hoechst 33342. Images present transmission light channel merged with blue fluorescence of cell nuclei (Hoechst 33342) and red fluorescence of A647-AnOs. Scale bar = 50 µm.

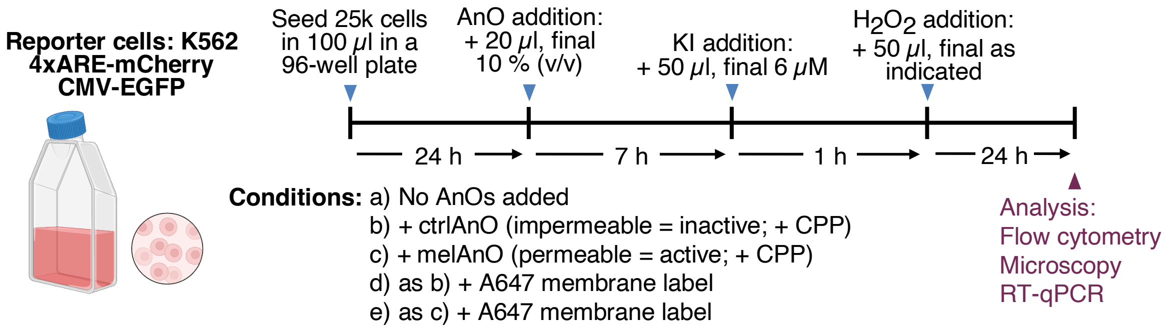

Figure S6. Experimental timeline of treating reporter cells with AnOs and required substrates. Reporter cells were seeded at 25,000 100 μl^{-1} per well and AnOs were added after 24 h. Non-fluorescent AnOs or A647-labelled AnOs with CPPs were supplemented as indicated. Medium with potassium iodide as electron donating co-substrate was administered 7 h after AnO supplementation, and H_2O_2 was added after an additional hour. 24 h after the oxidative insult with H_2O_2, cells were analyzed by flow cytometry, microscopy and/or RT-qPCR to check for NRF2 activation state and cell viabilities.

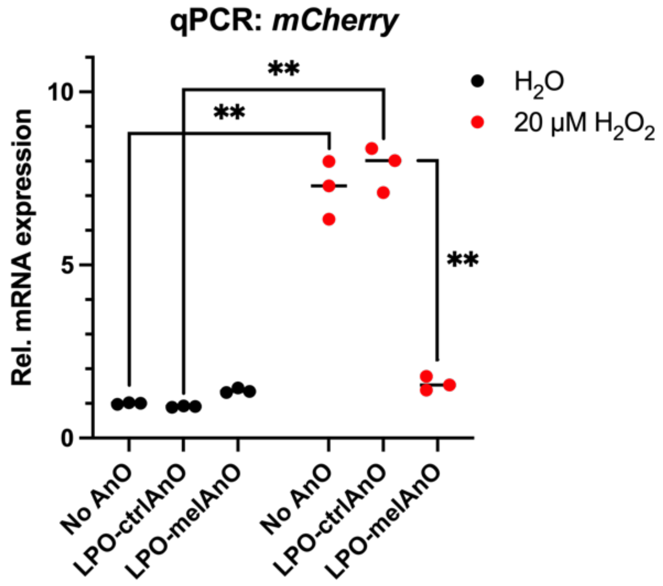

Figure S7. Dampened NRF2 transcriptional activation by scavenging intracellular H_2O_2 with AnOs (RT-qPCR of mCherry). mCherry mRNA levels were quantified by RT-qPCR in technical triplicates for two independent experiments (one repeat is shown). Statistical significance of differential expression was analyzed using a paired t-test in Prism (ns $P > 0.05$, * $P \leq 0.05$, ** $P \leq 0.01$, *** $P \leq 0.001$).

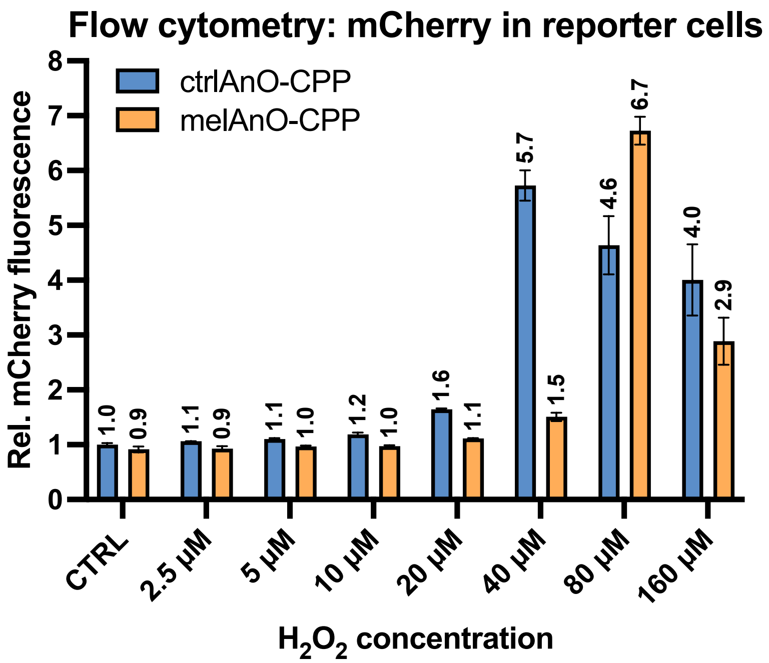

Figure S8. H_2O_2 scavenging by AnOs reduces NRF2 driven mCherry expression in a dose dependent manner (rel. mCherry levels). Cells were seeded as described and non-labelled AnOs (+ CPPs) were supplemented. NRF2 activation (mCherry fluorescence) was examined for increasing H_2O_2 concentrations with flow cytometry in duplicates (each data point 2x *N* = 5000 events; error bars = SD). Median of rel. mCherry fluorescence values are shown for each condition.

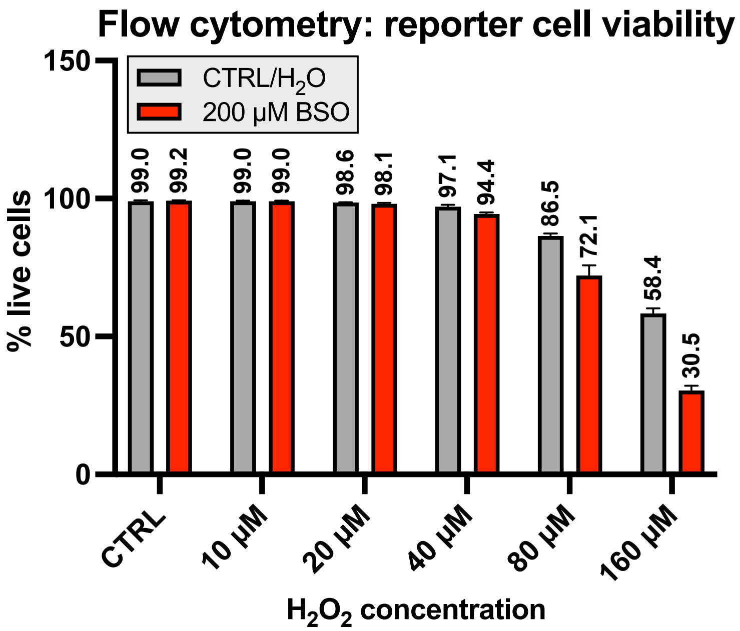

Figure S9. Reduced glutathione increases susceptibility to oxidative cell death (cell viabilities). Reporter cells were seeded and pre-treated with 200 µM BSO and subjected to increasing levels of H_2O_2 (without or with 10 µM, 20 µM, 40 µM, 80 µM, 160 µM H_2O_2). Cell viabilities of untreated vs. BSO-treated conditions (total viabilities) were determined by flow cytometry (in 4 replicates; error bars = SD).

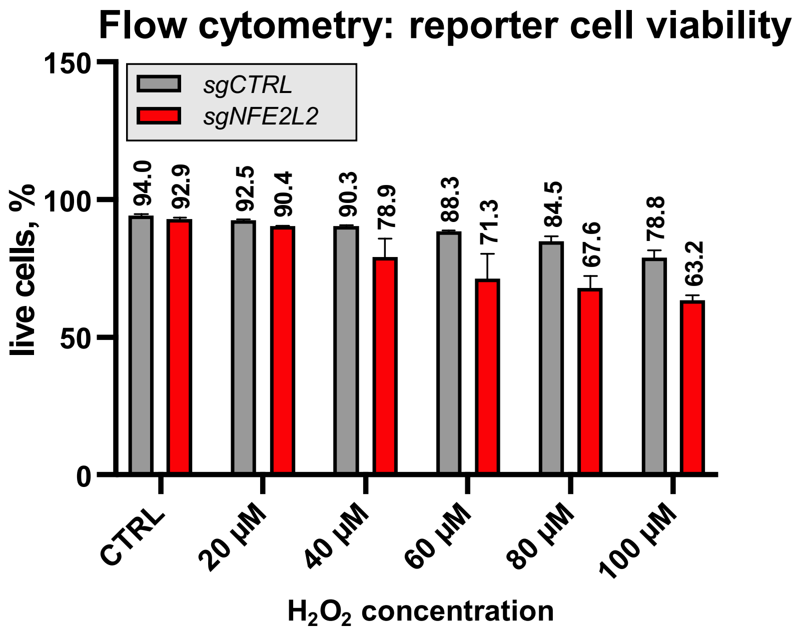

Figure S10. Knockdown of NRF2 causes reduced target gene expression and lower ROS defense (cell viabilities). The gene of NRF2 (*NFE2L2*) was mutated in reporter cells by sgRNA-guided CRISPR-Cas9-induced DNA cleavage. Reporter cells transduced with a nontargeting sgRNA served as control (*sgCTRL*). Reduced resilience against oxidative stress in the NRF2 knockdown was determined by flow cytometry (total viabilities) as before (in triplicates; error bars = SD).

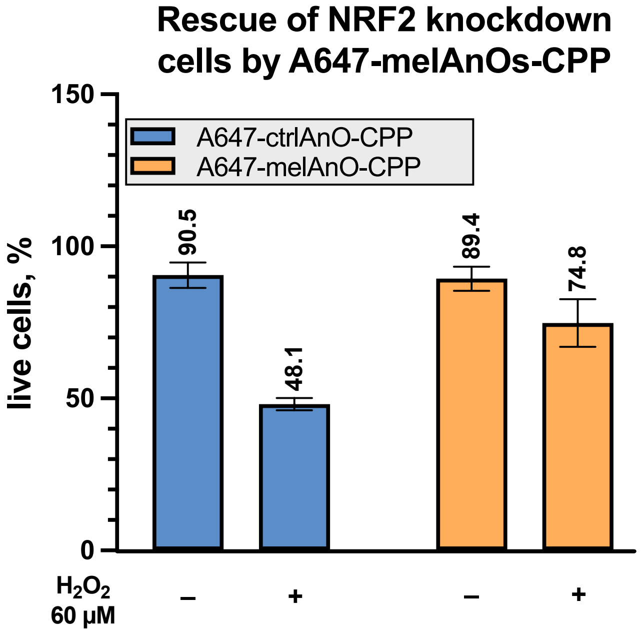

Figure S11. A647-melAnOs-CPP confer protection against oxidative stress in reporter cells with depleted NRF2 (cell viabilities). Reporter cells were stressed using 60 µM H_2O_2 and protection by functional A647-melAnOs-CPP was determined by quantifying dead cells with flow cytometry (in triplicates; error bars = SD).

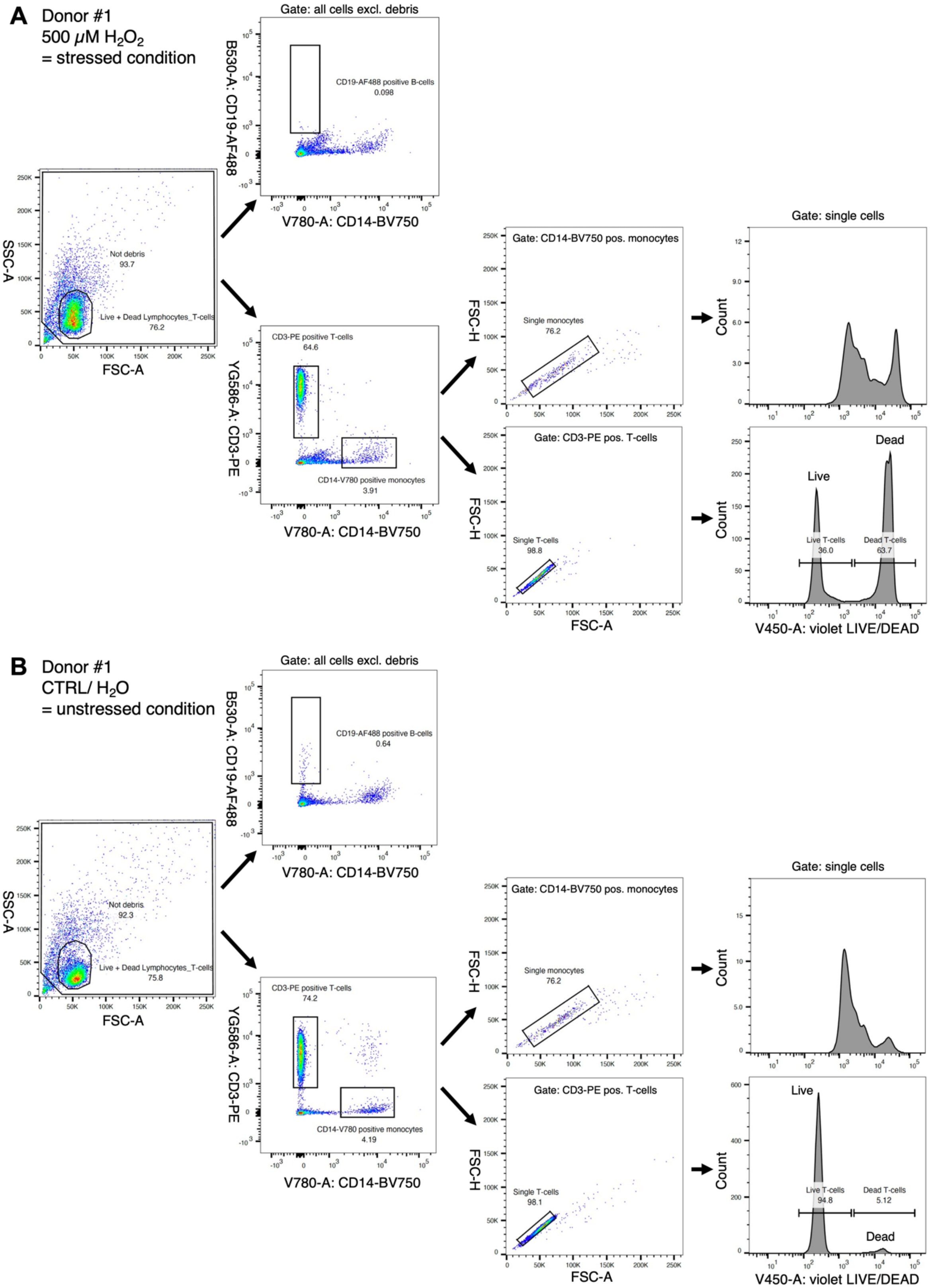
A
Donor #1
500 µM H2O2
= stressed condition
SSC-A
FSC-A
Not debris
93.7
Live + Dead Lymphocytes_T-cells
76.2
Gate: all cells excl. debris
B530-A: CD19-AF488
CD19-AF488 positive B-cells
0.098
V780-A: CD14-BV750
YG586-A: CD3-PE
CD3-PE positive T-cells
64.6
CD14-V780 positive monocytes
3.91
V780-A: CD14-BV750
Gate: CD14-BV750 pos. monocytes
FSC-H
Single monocytes
76.2
Gate: CD3-PE pos. T-cells
FSC-H
Single T-cells
98.8
FSC-A
Gate: single cells
Count
Live
Dead
Live T-cells
36.0
Dead T-cells
63.7
V450-A: violet LIVE/DEAD
B
Donor #1
CTRL/ H2O
= unstressed condition
SSC-A
FSC-A
Not debris
92.3
Live + Dead Lymphocytes_T-cells
75.8
Gate: all cells excl. debris
B530-A: CD19-AF488
CD19-AF488 positive B-cells
0.64
V780-A: CD14-BV750
YG586-A: CD3-PE
CD3-PE positive T-cells
74.2
CD14-V780 positive monocytes
4.19
V780-A: CD14-BV750
Gate: CD14-BV750 pos. monocytes
FSC-H
Single monocytes
76.2
Gate: CD3-PE pos. T-cells
FSC-H
Single T-cells
98.1
FSC-A
Gate: single cells
Count
Live
Live T-cells
94.8
Dead T-cells
5.12
Dead
V450-A: violet LIVE/DEAD

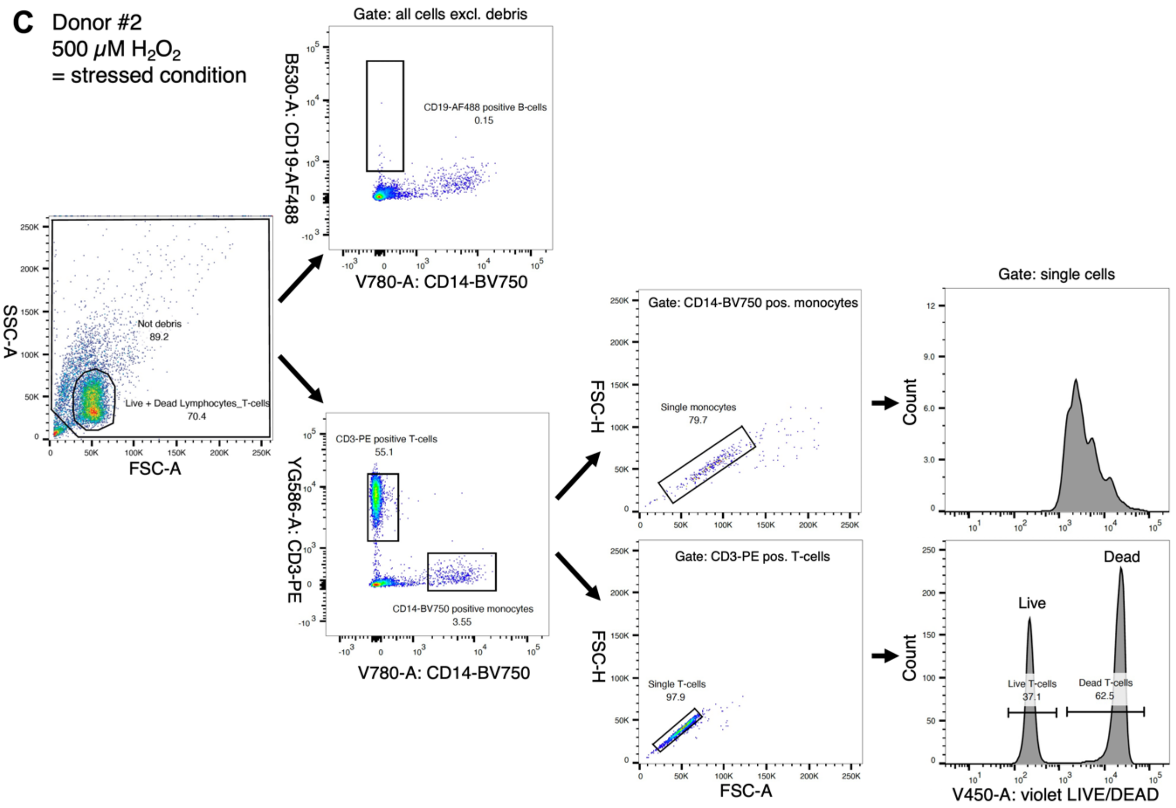
C
Donor #2
500 μM H2O2
= stressed condition
Gate: all cells excl. debris
B530-A: CD19-AF488
CD19-AF488 positive B-cells
0.15
V780-A: CD14-BV750
SSC-A
Not debris
89.2
Live + Dead Lymphocytes_T-cells
70.4
FSC-A
YG586-A: CD3-PE
CD3-PE positive T-cells
55.1
CD14-BV750 positive monocytes
3.55
V780-A: CD14-BV750
Gate: CD14-BV750 pos. monocytes
FSC-H
Single monocytes
79.7
Gate: single cells
Count
Gate: CD3-PE pos. T-cells
FSC-H
Single T-cells
97.9
FSC-A
Live
Dead
Count
Live T-cells
37.1
Dead T-cells
62.5
V450-A: violet LIVE/DEAD

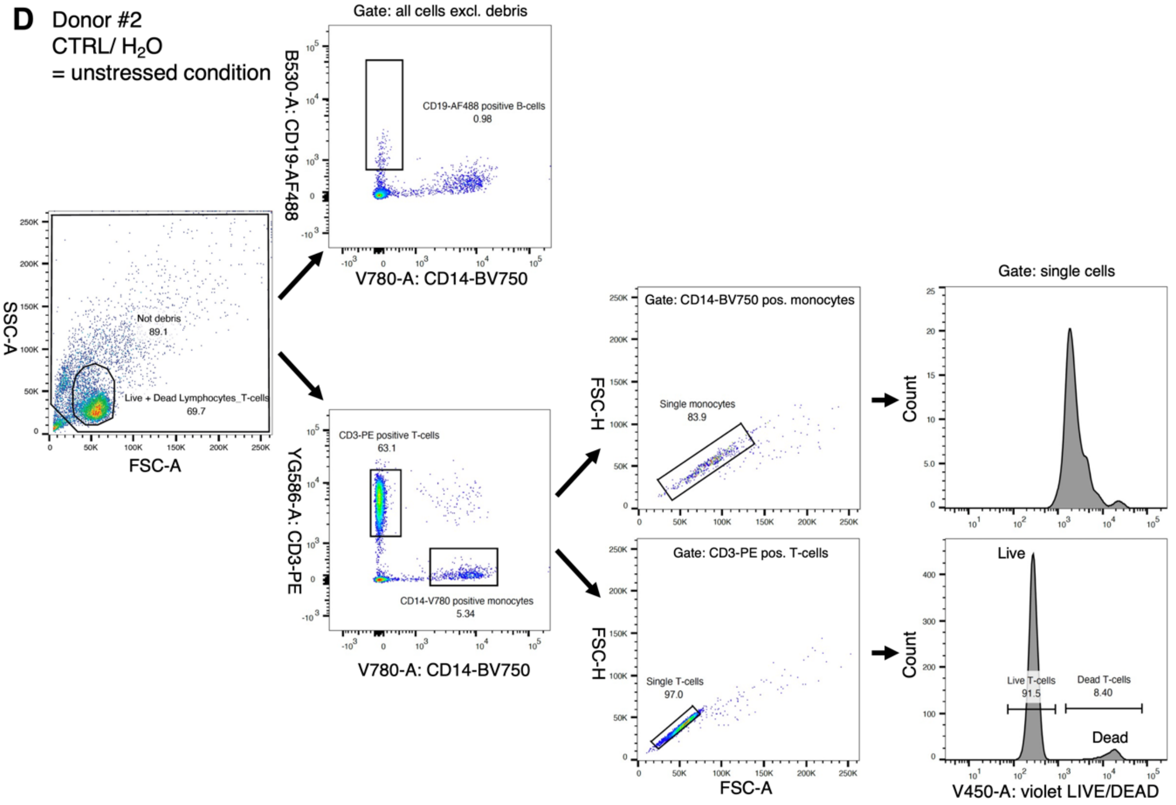
D
Donor #2
CTRL/ H2O
= unstressed condition
Gate: all cells excl. debris
B530-A: CD19-AF488
CD19-AF488 positive B-cells
0.98
V780-A: CD14-BV750
SSC-A
Not debris
89.1
Live + Dead Lymphocytes_T-cells
69.7
FSC-A
YG586-A: CD3-PE
CD3-PE positive T-cells
63.1
CD14-V780 positive monocytes
5.34
V780-A: CD14-BV750
Gate: CD14-BV750 pos. monocytes
FSC-H
Single monocytes
83.9
Gate: single cells
Count
Gate: CD3-PE pos. T-cells
FSC-H
Single T-cells
97.0
FSC-A
Live
Dead
Count
Live T-cells
91.5
Dead T-cells
8.40
V450-A: violet LIVE/DEAD

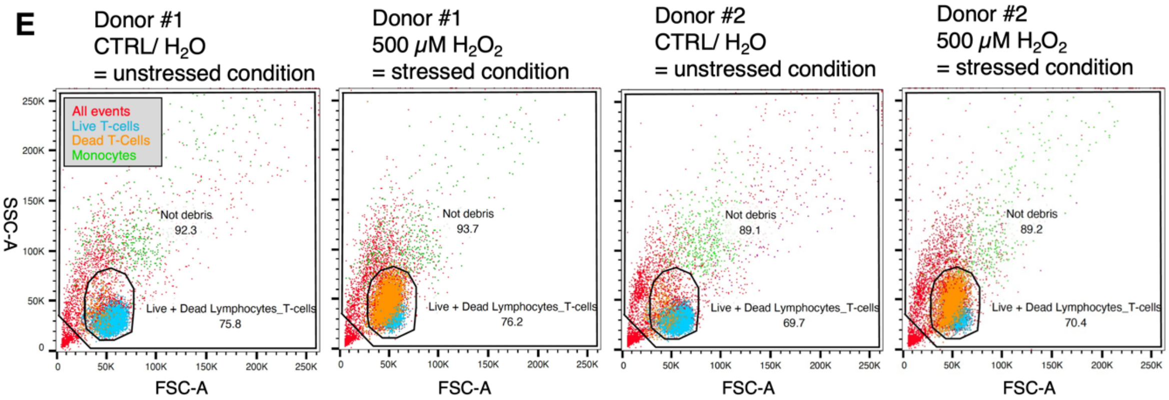

Figure S12. Identification of immune cell subpopulations in PBMCs under basal (= no H_2O_2) and stressed conditions (= 500 µM H_2O_2) using flow cytometry.

PBMCs from donor #1 and #2 were seeded at a density of 100,000 in 100 µl per well and either oxidative stress was induced by addition of 500 µM H_2O_2 (**A** donor #1, **C** donor #2) or cells were kept under basal conditions (**B** donor #1, **D** donor #2) for 24 h before analysis by flow cytometry. A total of N = 10,000 events (excluding debris) was analyzed for each condition. Debris was excluded by stringent gating in FSC-A:SSC-A plots and the T-cell, monocyte and B-cell subpopulations were identified by staining for CD3, CD14 and CD19 using tagged antibodies (CD3-PE, CD14-BV750, CD19-AF488). The percentage of B-cells was less than 1 % of all cells which is why we excluded them from further analysis. For the T-cell and monocyte subpopulation single cells were gated in FSC-H:FSC-A plots and violet LIVE/DEAD stain was used to determine percentages of live and dead cells. The violet LIVE/DEAD stain could only discriminate between live and dead T-cells, as for monocytes peak separation was not achieved. Live T-cells (blue), dead T-cells (orange) and total monocytes (green) were overlayed in FSC-A:SSC-A plots (**E**) to determine a gate for live & dead T-lymphocytes which was used for further analyses.

Supplementary Materials & Methods

Materials

DyLight 633 NHS ester, Amplex Red Reagent and enhanced Pierce bicinchoninic acid (BCA) assay were purchased from ThermoFisher Scientific (USA). Lactoperoxidase (LPO, from bovine milk), melittin (mel, from honey bee venom), proteinase K (from *Tritirachium album*), Sepharose® (4B, 45-165 µm beads diameter), and Whatman® Nucleopore™ Track-Etched membranes (100 nm) were purchased from Sigma-Aldrich (USA). Gibco™ RPMI 1640 medium, Gibco™ DMEM high glucose with pyruvate, and penicillin-streptomycin were purchased from ThermoFisher Scientific (USA). Fetal bovine serum (FBS) was purchased from Biowest (France).

Technical details for "Preparation of CPP-functionalized fluo-AnOs"

The dried polymer concentration was 10 mg mL^{-1} with both dyes, DyLight 633 NHS ester or Atto647N DOPE, at a concentration of 2 µM. After rehydration in PBS, resulting fluo-AnOs were extruded with an Avanti mini-extruder (Avanti Polar Lipids, Alabama, USA) through a polycarbonate (PC) membrane with a 100 nm diameter pore size for 11 times. Non-embedded fluorescent dye was separated from polymersomes by size exclusion chromatography (SEC, Sepharose 2B column; 37 cm length). Then, 1 µL of the DBCO-coupled CPP (7 mM in PBS) was added to 1 mL of the LPO-AnO suspension and stirred at 37 °C for 16 h in the dark.

Technical details for "AnO characterization by static (SLS) and dynamic light scattering (DLS)"

Multi-angle light scattering data were recorded on a spectrometer (LS Instruments, Switzerland) equipped with a 21 mW 633 nm He–Ne laser. All experiments were measured at scattering angles between 40 and 135° at 25 °C in round-bottom cuvettes (10 x 0.9–1.0 mm, Boro 3.3, LS Instruments (Fribourg, Switzerland). For both static (SLS) and dynamic (DLS) light scattering measurements, self-assembly dispersions diluted to 0.05 mg mL^{-1} were used without filtration. Hydrodynamic radii (R_h) were obtained from DLS measurements from the mean of three independent measurements over the whole range of angles using second order cumulant analysis. Polydispersity indices (PDI) were calculated from the 90° DLS measurements. For SLS analyses, mean intensities (N = 3) were plotted against the respective angle and fitted with a Mie scattering model (MiePlot, UK) for $\eta = 1.35$ and 5 % polydispersity. The radius R obtained from the best fit was transformed into the radius of gyration (R_g) using the formula for spherical structures $R_g^2 = (3/5)R^2$.

Technical details for "Transmission electron microscopy (TEM)"

For TEM, aliquots of AnOs in PBS at 0.25 mg mL^{-1} were adsorbed to a freshly glow-discharged, carbon-coated, parlodion-(2 % in n-butyl acetate) copper grid (Quantifoil, Germany). Then, the grid was washed four times with 50 µL water. After blotting excess liquid, the specimen was negatively stained with a 2% uranyl acetate solution (5 µL) for 10 s, washed three times with water and dried by blotting. TEM images were recorded on a CM100 transmission electron microscope (Philips, Eindhoven, The Netherlands) at an acceleration voltage of 80 kV.

Technical details for "Fluorescence correlation spectroscopy"

FCS experiments were conducted at 20 °C using a 40x water immersion objective (C-Apochromat 40x, NA 1.2; Carl Zeiss, Jena, Germany). For each measurement, 10 µL of sample were placed on a 22 × 50 mm glass slide, and a 633 helium/neon laser was used to excite DyLight 633. The laser was passed through the MBS488/561/633 filter and the signal was detected in the range of 657-690 nm with a pinhole aperture of 39 µm. Fluorescent fluctuations over time were recorded for 30 x 5 s. The raw data was processed and analyzed using ZEN software.

Enzyme quantification by bicinchoninic acid assay

The LPO encapsulation efficiency in AnOs was calculated using the enhanced Pierce bicinchoninic acid (BCA) assay according to the supplier's protocol (#23227, Thermo Scientific). AnOs were first ruptured by sonication and then incubated with ethanol at a ratio of 3:1 (v/v; final volume: 200 µL) at 37 °C for 1 h. The solution was passed through a 0.2 µm nylon membrane filter device (Whatman) and mixed with the BCA assay reagent at a 1:2 ratio (v/v). LPO standards and samples were incubated for 2 h at 37 °C, then the absorbance was measured at 562 nm using a SpectraMax id3 plate reader.

Technical details for "LPO enzymatic assay"

20 µL of LPO-AnOs (permeabilized and non-permeabilized control), 176 µL PBS, 2 µL H_2O_2 (200 µM) and 2 µL of AR (100 µM) were added to each well (final volume: 200 µL) of a black 96-well microplate and monitored for 20 min at RT in a Spectramax id3 plate reader (λ excitation 568 nm, λ emission 581 nm). Each experimental condition was carried out in triplicates.

Culturing K562 cells

K562 leukemia cells (received from ATCC) and their derivatives were cultured in Roswell Park Memorial Institute 1640 medium (RPMI, #21875034, Gibco^{TM} ThermoFisher Scientific), supplemented with 10 % (v/v) fetal bovine serum (FBS, #S181H-50, Biowest VWR) and 1 % (v/v) penicillin-streptomycin (#15140122, Gibco^{TM} ThermoFisher Scientific) at 37 °C and 5% CO_2 in a humidified incubator. K562 cultures were routinely maintained at concentrations between 0.25 x 10^6 and 1 x 10^6 cells mL^{-1}. For ensuring sterility during AnO addition, gentamycin was optionally added (#L0011-100, Biowest).

Culturing HEK293T cells

HEK293T cells (received from ATCC) were cultured in Dulbecco's modified Eagle's medium containing high glucose and pyruvate (DMEM, #41966029, GibcoTM ThermoFisher Scientific), supplemented with 10 % (v/v) FBS and 1 % (v/v) penicillin-streptomycin at 37 °C and 5 % CO_2 in a humidified incubator. HEK293T cells were routinely subcultured every 2-3 day at a ratio of 1:5.

Culture of peripheral blood mononuclear cells (PBMCs)

Buffy Coats were obtained from Blutspende SRK Zurich. A total of two separate healthy donors participated in this study. PBMC medium consisted of PRMI 1640 medium with GlutaMAX™ and HEPES buffer (#7240021, Gibco^{TM} ThermoFisher Scientific) which we further supplemented with 10 % (v/v) FBS, 1 % (v/v) penicillin-streptomycin, 1 % (v/v) MEM non-essential amino acids solution (#11140035, Gibco^{TM} ThermoFisher Scientific), 1 mM sodium pyruvate (#11360070, Gibco^{TM} ThermoFisher Scientific) and 50 µM β-mercapto-ethanol (#31350010, Gibco^{TM} ThermoFisher Scientific). Medium was refreshed twice per week by pelleting cells by centrifugation at 300 g for 5 min and resuspension at a concentration of 1 x 10^6 cell mL^{-1} in PBMC medium. PBMCs were cultured at this concentration in a humidified incubator at 37 °C and 5 % CO_2.

Technical details for "Lentiviral infections"

For lentivirus production, 1.8 x 10^6 HEK293T cells were seeded in 4 mL DMEM in a 6 cm culture dish 24 h prior to transfection. 200 µL serum-free DMEM were mixed with 1 µg of the cloned plentiCRISPR-v2 plasmid, 0.1 µg pCMV-VSV-G plasmid (#8454, Addgene), 0.9 µg psPAX2 plasmid (#12260m Addgene) and 6 µL of X-tremeGENE^{TM} HP DNA transfection reagent (#6366244001, Roche/Sigma-Aldrich) and incubated for 20 min at RT. Subsequently, the transfection mix was added dropwise onto the HEK293T cells. After 48 h incubation at 37 °C and 5 % CO_2, the virus-containing supernatant was

harvested and passed through a 0.45 µm filter. 1 x 10^6 reporter cells suspended in 2 mL RPMI medium were infected with 1 mL of purified lentivirus in the presence of 2 µL of polybrene (#TR-1003-G, Merck). Lentiviral supernatant was removed 48 h after infection by centrifugation (160 g, 3 min) and cells were reconstituted in fresh RPMI culture medium. Cells infected with lentivirus for NRF2 knockdown, or the non-targeting control, were selected with 5 µg mL^{-1} blasticidin (#15205-25MG, Sigma-Aldrich) for ten days.

psPAX2 was kindly supplied by Didier Trono (Addgene#12259), and pCMV-VSV-G was a gift from Bob Weinberg (Addgene plasmid # 8454; http://n2t.net/addgene:8454; RRID:Addgene_8454), lentiCRISPR-v2 was a gift from Feng Zhang (Addgene plasmid # 52961 ; http://n2t.net/addgene:52961 ; RRID:Addgene_52961).

Technical details for "Isolation of PBMCs from buffy coats"

For isolating immune cells from blood, buffy coats were diluted ~ 1:4 in PBS and 15 mL lymphoprep™ density gradient medium (#07851, STEMCELL Technologies) was filled per SepMate tube (#85450, STEMCELL Technologies) before 35 mL of diluted buffy coat was added without disturbing the density gradient medium. Blood cells were separated by centrifugation at 1,200 g for 10 min and the PBMC containing top layer was harvested. The PBMC fraction from several SepMate tubes were pooled and pelleted by centrifugation at 120 g for 10 min (no break) and then washed by resuspension in 50 mL PBS followed by centrifugation at 300 g for 8 min. For freezing, PBMCs were normalized to 100 x 10^6 mL^{-1} after centrifugation at 300 g for 5 min and resuspended in FBS containing 10 % DMSO. Cells were frozen at - 80 °C and transferred the day after to liquid nitrogen for long term storage. For thawing PBMCs, PBMC aliquots were thawed at 37 °C in the presence of 250 µL 1 mg mL^{-1} DNAse (#DN25-100mg, Sigma-Aldrich) to avoid precipitates.

Technical details for "NRF2 activity assay for K562 reporter cells using flow cytometry"

Cell seeding:

Generally, reporter cells were seeded in 100 µL RPMI medium supplemented with 50 µg mL^{-1} gentamycin at 2.5 × 10^4 cells per well. If cells were pre-treated with BSO, cells were alternatively seeded in 50 µL RPMI medium and treated 6 h later with 50 µL 2x BSO in RPMI resulting in 200 µM BSO per well (BSO: #B2640-500MG, Sigma-Aldrich). After 24 h of culture, 20 µL of AnOs in PBS were added to the cells for a final AnO concentration in the well of 10 % (v/v). After 7 h incubation, 50 µL RPMI culture medium containing a 4x KI concentration (#207969-100G, Sigma-Aldrich) were added to each well resulting in a final KI concentration of 6 µM. After 1 h of KI exposure, cells were treated with 50 µL RPMI culture medium containing H_2O_2 (H_2O_2 solution purum p.a., ≥35%, Sigma-Aldrich, #95299-500ML) at 4x the concentration of the final concentration per well (total medium volume 200 µL). After 24 h incubation with H_2O_2, cells were examined by flow cytometry or microscopy, or RNA was extracted for performing RT-qPCR.

Processing of cells for flow cytometry:

In preparation for flow cytometry, non-internalized AnOs were washed away by repeated centrifugation and resuspension steps, and dead cells were labelled with violet live/dead™ stain (#L34963, Thermo Scientific) according to the supplier's protocol. After AnO and/or H_2O_2 treatment, cells were harvested by centrifugation (160 g, 3 min) in U-bottom 96 well plates and washed in 1x 200 µL PBS by resuspension and centrifugation (160 g, 3 min). For labelling dead cells, cell pellets were resuspended in 100 µL violet live/dead™ dye in DMSO, diluted 1:1000 in PBS, and incubated for 30 min at RT in the dark. The labelling reaction was stopped by adding 100 µL FACS buffer (PBS with 1% bovine serum albumin from PAN Biotech, #P06-1391100, 2 mM EDTA from Sigma, #E6511) and cells were washed 2x with 200 µL FACS buffer by centrifugation (160 g, 3 min) and resuspension.

Acquisition details for flow cytometry:
Dead cells were quantified with the live/dead™ stain. K562 reporter cells expressed GFP under control of a constitutive CMV promotor to report global protein expression and mCherry in response to NRF2 activation. Uptake of AnOs in K562 reporter cells was investigated by either quantifying DL633 or A647 fluorescence. Per data point a total of 5,000 events was recorded.

Cells were analyzed at a flow speed of 1 µL s^{-1} with the following cytometer settings:
Violet live/dead™ stain: excitation (ex.) at 405 nm, emission (em.) BP Filter 450/40, no LP mirrors
GFP: excitation ex. 488 nm, em. BP Filter 530/30, LP mirrors 505
mCherry: ex. 561 nm, em. BP Filter 610/20, LP mirrors 600
DL633/A647: ex. 640 nm, em. BP Filter 670/30, no LP mirrors

Analysis details for calculating NRF2 activities and AnO uptake:
Flow cytometry standard files were exported from BD LSRFortessa DIVA software and analyzed in FlowJo using hierarchical gating: 1) stringent, polygonal gate framing the live-cell population in FSC-A:SSC-A plots while excluding dead cells and debris; 2) diagonal, rectangular gate in FSC-H:FSC-A plots to exclude doublets; 3) GFP-A intensity histogram plot: two populations were detected and GFP-negative cells were excluded from further analysis.
Means of GFP intensities of GFP positive cells, means of mCherry intensities and DL633 or A647 median fluorescent intensities were exported as table from FlowJo and further analyzed in Microsoft Excel. DL633/A647 intensity histograms were plotted in FlowJo using the layout editor.
For NRF2 activity assessment, mCherry intensity medians were divided by GFP intensity medians for each sample and fold changes were calculated by normalizing to the respective control condition. Results were plotted in Prism (V9.2.0).

Analysis details for calculating cell viabilities:
Debris was excluded by polygonal gating in FSC-A:SSC-A plots while conserving dead cells and two populations were detected in violet live/dead™ histograms: live and dead cells with low or high violet live/dead™ intensity, respectively. Histogram gating was used to demarcate dead cells (= high violet live/dead™ intensity) from live cells to determine cell viabilities. Data were exported and analyzed in Microsoft Excel to calculate the percentage of living cells per condition and data were plotted in Prism (V9.2.0).

Technical details for: "Killing and rescue assay with PBMCs using flow cytometry"
Cell seeding:
PBMCs were seeded in 100 µL PBMC medium at 10^5 cells per wells. AnO supplementation, as well as KI and H_2O_2 were identical as for K562 reporter cells, but in PBMC medium. After 24 h incubation with H_2O_2, PBMCs were processed for flow cytometry and/or microscopy. PBMCs were prepared for flow cytometry as K562 reporter cells. Uptake of AnOs in T-lymphocytes was quantified by measuring A647 fluorescence.

PBMC viability assessment and gating for T-lymphocytes:
To gate the live and dead T-lymphocyte populations for later analyses, PBMCs were stained for CD3, CD14 and CD19 using conjugated antibodies. Conjugated antibodies were purchased from BioLegend (PE-CD3: #981004; BV750-CD14: #367135, AF488-CD19: #363037). PBMCs were washed in 1x 200 µL FACS buffer by centrifugation and resuspension (300 g, 3 min). Fc receptors on PBMCs were blocked with 100 µL Human TruStain FcX™ (#422301, BioLegend) diluted 1:20 in FACS buffer for 10 min at RT. PBMCs were washed by centrifugation (300 g, 3 min) and

resuspension in 1x 200 µL FACS buffer. PBMCs were then stained for CD3, CD14 and CD19 by antibodies diluted 1:100 in FACS buffer for 30 min at 4°C in the dark. PBMCs were washed by adding 100 µL PBS followed by centrifugation (300 g, 3 min) and resuspension in 1x 200 µL PBS followed by another centrifugation (300 g, 3 min). Dead cells were labelled with violet live/dead™ stain (diluted 1:1000 in PBS) for 30 min at RT in the dark and the reaction was stopped by adding 100 µL FACS buffer followed by centrifugation (300 g, 3 min). PBMCs were washed again with FACS buffer as before. Cells were then fixed in 100 µL 4 % formaldehyde (#P087.5, Carl Roth) for 10 min at RT in the dark and the reaction was stopped by addition of 100 µL FACS buffer followed by centrifugation (300 g, 3 min). PBMCs were washed again with FACS buffer as before. Cells were resuspended in 200 µL FACS buffer and analyzed by flow cytometry.

PBMCs were analyzed on a BD LSRFortessa and fluorochrome spillover was compensated using single-stained controls (for antibodies: CompBead Plus #560497, Becton Dickinson; for live/dead stain: Arc Amine Reactive Compensation Bead Kit, #A10628, Thermo Fisher) prepared according to the instructions of the manufacturer.

Acquisition details for flow cytometry:
Cells were analyzed at a flow speed of 1.5 µL s^{-1} with the following cytometer settings:
Violet live/dead™ stain: ex. at 405 nm, em. BP Filter 450/40, no LP mirrors
BV750-CD14: ex. at 405 nm, em. BP Filter 780/60, LP mirrors 735
AF488-CD19: ex. at 488 nm, em. BP Filter 530/50, LP mirrors 505
PE-CD3: ex. at 561 nm, em. BP Filter 586/15, LP mirrors 570
A647: ex. 640 nm, em. BP Filter 670/30, no LP mirrors

Technical details for RT-qPCR
For gene expression analysis in reporter cells, RNA was extracted using the RNeasy Plus Mini Kit (#74136, Qiagen) according to the instructions of the manufacturer. 500 ng of RNA were reverse transcribed to cDNA using the iScript™ Advanced cDNA Synthesis Kit (#1725038, BioRad) according to supplier's protocols. Then, gene expression was analyzed by RT-qPCR in technical triplicates on a CFX Connect™ Optics Module (BioRad) light cycler using SYBR master mix (#1725271, BioRad) according to the manufacturer's instructions. Primers were purchased from Microsynth.

Per well of a Hard-Shell® 96-Well PCRHSP9655 Plate (#HSP9655, Biorad), we mixed 10 µL of 2 x SYBR master mix (#1725271, BioRad), 4 µL of diluted cDNA, 2 µL each of forward and reverse primer, and 2 µL RNAse/DNAse free H_2O. The plate was sealed with Microseal® B Adhesive Sealers (#MSB-1001, BioRad) and the qPCR reaction was observed on a CFX Connect™ Optics Module (BioRad) light cycler over a total of 40 cycles after 3 min denaturation at 95 °C: a) 10 s at 95 °C, b) 30 s at 62 °C, melting curve: increase of 0.5 °C every 5 s until 95 °C. Primer dimers were excluded by melting curve analysis.

1) 18S ribosomal RNA (fw: TGTGCCGCTAGAGGTGAAATT, rv: TGGCAAATGCTTTCGCTTT)
2) NQO1 (fw: GAAGAGCACTGATCGTACTGGC, rv: GGATACTGAAAGTTCGCAGGG)
3) OSGIN1 (fw: AACCCCATTGACGTGGACC, rv: CAAACCTCACGAAGTTGTCCC)
4) mCherry (fw: GAACGGCCACGAGTTCGAGA, rv: CTTGGAGCCGTACATGAACTGAGG)

C_q values were exported as XLSX files and analyzed in Microsoft Excel. Relative mRNA levels were calculated using the $2^{-\Delta\Delta Ct}$ method and fold changes were calculated by first normalizing to 18S rRNA levels and further to the untreated controls. Results were tested for statistical significance (paired t-test) and plotted in Prism (V9.2.0).

Technical details for western blotting

Cell seeding, treatments & lysis

For determining protein levels, reporter cells were seeded in 1.2 ml at 0.25 $x10^{-6}$ cells mL^{-1} in a 6-well plate (#Z707759, TPP) and treated without or with 20, 40, 60, 80 or 100 µM H_2O_2 for 24 h. After the incubation time, cells were pelleted and snap frozen in liquid nitrogen. After defrosting in lysis buffer (1 % Triton-X-100, #437002A, VWR; cOmplete™ Protease Inhibitor Cocktail, #11836170001, Sigma-Aldrich; 40 mM HEPES, #3724, Applichem; 5 mM $MgCl_2$, #63068-250G, Sigma-Aldrich; 10 mM KCl, #60130-250G, Sigma-Aldrich), cells were homogenized by pipetting and supersonication for 20 min at 4 °C in a cooled waterbath and subsequently inverted in an end-over-end shaker for 30 min at 4 °C to complete lysis. Lysate was pre-cleared by centrifugation (14,000 g, 15 min, 4 °C) and purified supernatant was harvested.

SDS-PAGE and wet transfer

Protein concentrations in cell lysates were determined with Bradford reagent (#5000006, Biorad) and then normalized to the lowest concentration. Lysates were heat-denatured by incubation at 95 °C for 5 min. Proteins were separated by SDS-PAGE (running buffer prepared as 10X: 1 L H_2O + 30 g Trizma® base, #T1503-1KG, Sigma-Aldrich; + 144 g Glycine, #G7126-1KG, Sigma-Aldrich; + 10 g SDS, #A4159, Applichem). Proteins were transferred onto Immun-Blot® PVDF Membranes (#162-0177, BioRad) by wet transfer (transfer buffer: 1.8 L H_2O + 200 mL ethanol abs. + 4.44 g CAPS, #C2632-100G, Sigma-Aldrich, + 10 tablets NaOH, #28244.295, VWR).

Incubation with primary and secondary antibody + chemiluminescent readout

After wet transfer, membranes were blocked by immersing in 5 % (m/v) milk (Rapilait, #L30195806, Migros) diluted in TBST (prepared as 10X: 1 L H_2O + 24 g Trizma® base; + 88 g NaCl, #S5886-500G, Sigma-Aldrich; + 1 % (v/v) Tween20, #P1379-500ML, Sigma-Aldrich) for 15 min. Membranes were probed with primary antibodies (1:1000 NRF2, # ab62352, Abcam; 1:1000 mCherry, # ab167453, Abcam; 1:1000 β-Actin, # ab8226, Abcam; 1:1000 NQO1, #3187S, CST), while rocking at 4 °C over night. Membranes were washed 3x with TBST. Membranes were then probed with secondary antibody (1:5000 Peroxidase AffiniPure Donkey Anti-Rabbit IgG, #711-035-152; 1:5000 Peroxidase AffiniPure Donkey Anti-Mouse IgG, #715-035-150; both Jackson ImmunoResearce; in 20 ml 5 % (v/v) milk in TBST) for 1 h at RT on a rocker plate. Membranes were washed again as described above. For the chemiluminescent readout, membranes with mCherry, β-Actin and NQO1 were treated with WesternBright™ ECL substrate (#K-12045-C20, Advansta) and membranes with NRF2 were treated with WesternBright™ Sirius (#K-12043-C20, Advansta). Chemiluminescence was determined on a Fusion FX (Vilber) readout system.

Technical details for: "Quantification of glutathione"

For determining cellular glutathione levels, K562 reporter cells were seeded in 1.2 mL PRMI medium at a concentration of 0.5 x 10^6 cells mL^{-1} in transparent, flat-bottom 6-well microtiter plates (#Z707759, TPP). After 6 h, cells were treated without or with 200 µM BSO by adding 1.2 mL RPMI medium at a 2x BSO concentration. KI was added 24 h after seeding by supplementing 1.2 mL RPMI medium at a 4x concentration resulting in 6 µM per well and 1 h later cells were treated without or with 40 µM H_2O_2 in 1.2 mL RPMI medium also at 4x of the final concentration of the well. After 24 h, cells were harvested by centrifugation (160 g, 3 min) and washed 1x in 1 mL PBS. Cells were then further processed according to the instructions of the kit's manufacturer. The amount of chromogenic product was measured after 30 min in technical triplicates on a TECAN Spark 10M UV/VIS plate photometer at 405 nm.

Technical details for: “Fluorescence microscopy”

Details for deposition of cells by cytospin

K562 reporter cells or PBMCs were seeded and treated with labelled AnOs, KI and H_2O_2 as described for flow cytometry. After incubation, cells from 2-3 wells of the 96-well culture plate were pooled. Pooled cells were washed by resuspension in 200 µL PBS and centrifugation (300 g for 3 min). Cells were fixed in 200 µL 4 % formaldehyde (#P087.5, Carl Roth) for 10 min at RT in the dark and cells were harvested by centrifugation (300 g, 3 min). Cells were washed again with PBS buffer as before. Plasma membranes were stained with 200 µL CF®488A Wheat Germ Agglutinin (WGA488, #29022-11, Biotium) diluted 1:1000 in PBS for 15 min at RT in the dark and pelleted and washed as before. Finally, cells were resuspended in 50 µL PBS and deposited with cytofunnels (Fisherbrand™ Single Cytology Funnels, #10-354, Thermo Fisher Scientific) and filter cards (#5991022, Thermo Fisher Scientific) on cytoslides™ (#5991051, Thermos Andon) using a Shandon Cytospin 2 Centrifuge (Thermo Fisher Scientific) at 300 rpm for 3 min. After air drying the cell layer, cells were immersed in Prolong™ Glass Antifade Mountant with NucBlue™ Stain (#P36981, Thermo Fisher Scientific), which contained Hoechst stain for visualizing nuclei, and glass slides were sealed with nail polish.

Acquisition details for imaging with line scanning confocal microscope (Figure 3)

CPP-facilitated uptake of AnOs in reporter cells was examined on a Leica TCS SP8 setup operated by LASX software. 8-bit images were acquired using a HC PL APO CS2 63x/1.40 objective in confocal mode (pinhole 95.5 µm, 2.25x optical zoom, with 2x line average) with oil immersion as z-stacks (3 planes, range 2 µm, distance 1 µm) with 1024x1024 pixels which scales to 82x82 µm in size. Fluorophores were excited in the listed order:

DL633: HeNe 633 laser; 633 nm laser line at 35 % intensity; HyD 3 detector range 650-800 nm at gain 100.

WGA488: Argon laser (19.8 % intensity); 488 nm laser line at 1.2 % intensity; HyD 1 detector range 500-550 nm at gain 100.

Hoechst: Diode 405; 405 nm laser line at 6 % intensity; HyD 1 detector range 418-489 nm at gain 100.

Images were exported as tagged image file (TIF) and processed in Image J Version 2.9.0. Z-stacks were merged as maximum intensity projections. For improved visibility, channel look-up-tables (LUT) were changed and brightness/contrast was adjusted accordingly for all imaged ROIs (**DL633:** LUT “fire” min. 15 max. 140; **WGA488:** LUT “green” min. 5 max. 255; **Hoechst:** LUT “grays” min. 0 max. 255).

Acquisition details for imaging with spinning disk confocal microscope (Figure 4)

Simultaneous AnO uptake and NRF2 activation was imaged on a spinning disk microscope. The spinning disk setup comprised a Nikon Eclipse Ti2 (inverse) microscope body, a Confocal Scanner Unit CSU-W1-T2 SoRa with 50 µm pinholes at 500 µm spacing and a sCMOS Hamamtasu Orca Fusion BT camera (2304 x 2304 pixels, 6.5 x 6.5 µm pixel size). 12-bit images were acquired in sensitive mode as z-stacks (3 planes, range 2 µm, distance 1 µm) using a 60x objective NF2 1.40 NA with oil immersion. Fluorophores were excited in the listed order:

A647: 647 nm laser line at 100 % intensity; 1000 ms exposure; ET 708/75 filter.

mCherry: 561 nm laser line at 100 % intensity; 500 ms exposure; ET 630/75 filter.

WGA488: 488 nm laser line at 30 % intensity; 500 ms exposure; ET 525/50 filter.

Hoechst: 405 nm laser line at 30 % intensity; 200 ms exposure; ET447/60 filter.

Images were exported as ND2 files and processed in Image J Version 2.9.0. Z-stacks were merged as maximum intensity projections. For improved visibility, central regions of images were cropped (from pixels xy 220, 220 to xy 1850, 1850), LUTs were changed and brightness/contrast was adjusted accordingly for all imaged ROIs (**A647:** LUT “fire” min. 250 max. 800; **mCherry:** LUT “yellow” min.

125 max. 700; **WGA488:** LUT “green” min. 50 max. 2500; **Hoechst:** LUT “grays” min. 60 max. 2000).

Acquisition details for imaging with airyscan superresolution microscopy (Figure 6)
AnO uptake in PBMCs was examined on a Zeiss LSM 880 AxioObserver. 16-bit images were acquired as z-stacks (3 planes, range 2 µm, distance 1 µm) using a plan-apochromat 63x NA 1.40 DIC M27 objective with oil immersion with 1888x1888 pixels which scales to 67x67 µm in size. Fluorophores were excited in the listed order using a gain of 800 and a BP 495-550 + LP 570 filter for all channels:
A647: HeNe633 laser at 633 nm with 80 % intensity
WGA488: Argon laser at 458 nm with 10 % intensity
Hoechst: Diode 405-30 at 405 nm with 9 % intensity
Superresolution images were calculated in ZEN 2012 software using the 2D airyscan processing algorithm with constant processing strength of 7.3 to allow for comparable intensities between conditions. Images were exported as CZI files for further processing in Image J Version 2.9.0. Z-stacks were merged as maximum intensity projections and for improved visibility, central regions with cells of interest were cropped (from pixels xy 185, 185 to xy 1450, 1450). LUTs were changed and brightness/contrast was adjusted accordingly for all imaged ROIs (**A647:** LUT “fire” min. 50 max. 1500; **WGA488:** LUT “green” min. 20 max. 1500; **Hoechst:** LUT “grays” min. 0 max. 7000).
